# microRNA miR-142-3p Inhibits Breast Cancer Cell Invasiveness by Synchronous Targeting of WASL, Integrin Alpha V, and Additional Cytoskeletal Elements

**DOI:** 10.1371/journal.pone.0143993

**Published:** 2015-12-10

**Authors:** Alexander Schwickert, Esther Weghake, Kathrin Brüggemann, Annika Engbers, Benjamin F. Brinkmann, Björn Kemper, Jochen Seggewiß, Christian Stock, Klaus Ebnet, Ludwig Kiesel, Christoph Riethmüller, Martin Götte

**Affiliations:** 1 Department of Gynecology and Obstetrics, Münster University Hospital, Münster, Germany; 2 Institute-associated Research Group "Cell adhesion and cell polarity”, Institute of Medical Biochemistry, ZMBE, University of Münster, Münster, Germany; 3 Center for Biomedical Optics and Photonics, University of Muenster, Muenster, Germany; 4 Biomedical Technology Center, Medical Faculty, University of Münster, Münster, Germany; 5 Institute for Human Genetics, Medical Faculty of the University of Münster, Münster, Germany; 6 Institute of Physiology II, University of Münster, Münster, Germany; 7 Department of Gastroenterology, Hepatology and Endocrinology, Hannover Medical School, Hannover, Germany; 8 Center for Nanotechnology, Serend-ip GmbH, Münster, Germany; University of Patras, GREECE

## Abstract

MicroRNAs (miRNAs, micro ribonucleic acids) are pivotal post-transcriptional regulators of gene expression. These endogenous small non-coding RNAs play significant roles in tumorigenesis and tumor progression. miR-142-3p expression is dysregulated in several breast cancer subtypes. We aimed at investigating the role of miR-142-3p in breast cancer cell invasiveness. Supported by transcriptomic Affymetrix array analysis and confirmatory investigations at the mRNA and protein level, we demonstrate that overexpression of miR-142-3p in MDA-MB-231, MDA-MB-468 and MCF-7 breast cancer cells leads to downregulation of *WASL* (Wiskott-Aldrich syndrome-like, protein: N-WASP), Integrin-αV, *RAC1*, and *CFL2*, molecules implicated in cytoskeletal regulation and cell motility. *ROCK2*, *IL6ST*, *KLF4*, *PGRMC2* and *ADCY9* were identified as additional targets in a subset of cell lines. Decreased Matrigel invasiveness was associated with the miR-142-3p-induced expression changes. Confocal immunofluorescence microscopy, nanoscale atomic force microscopy and digital holographic microscopy revealed a change in cell morphology as well as a reduced cell volume and size. A more cortical actin distribution and a loss of membrane protrusions were observed in cells overexpressing miR-142-3p. Luciferase activation assays confirmed direct miR-142-3p-dependent regulation of the 3’-untranslated region of *ITGAV* and *WASL*. siRNA-mediated depletion of *ITGA*V and *WASL* resulted in a significant reduction of cellular invasiveness, highlighting the contribution of these factors to the miRNA-dependent invasion phenotype. While knockdown of *WASL* significantly reduced the number of membrane protrusions compared to controls, knockdown of *ITGAV* resulted in a decreased cell volume, indicating differential contributions of these factors to the miR-142-3p-induced phenotype. Our data identify *WASL*, *ITGAV* and several additional cytoskeleton-associated molecules as novel invasion-promoting targets of miR-142-3p in breast cancer.

## Introduction

MicroRNAs (miRNAs) are endogenous small non-coding RNAs comprised of approximately 19–25 nucleotides. Primary miRNA transcripts are cleaved by the RNase enzyme complex Drosha-DGCR8 in the nucleus and subsequently by the action of the cytoplasmic RNase III Dicer1 [1–3]. One miRNA duplex strand is degraded while the other strand is incorporated into the microRNA ribonucleoprotein complex which binds to partially complementary target sites in the 3’-untranslated region (3’UTR) of target mRNAs. Depending on the degree of complementarity, expression of the encoded protein is either repressed translationally or its mRNA is degraded [3,4]. miRNAs have emerged as regulators of gene expression in critical cellular processes such as differentiation, apoptosis and stem cell renewal [2, 3]. In breast cancer, dysregulated miRNA expression is associated with tumor occurrence, progression and metastasis [5–10]. miR-142-3p is dysregulated in clinical samples of breast cancer and breast cancer cell lines relative to normal breast tissue [8,11]. The functional role of miR-142-3p in breast cancer cells has not been elucidated, yet. However, miR-142-3p-dependent functions have been identified in hematopoiesis [12], the immune system [13] and in relation with hemato-oncological diseases [14]. Aberrant expression or function of miR-142-3p has been noted in several additional forms of cancer, including clear-cell kidney cancer, non-small cell lung cancer, hepatocellular carcinoma, colon cancer and thyroid cancer [15–18]. In this study, we aimed at investigating a potential role for miR-142-3p in the regulation of breast cancer invasiveness *in vitro*. We demonstrate that upregulated miR-142-3p expression levels in a panel of human breast cancer cell lines leads to inhibition of Integrin-αV, a transmembrane receptor subunit implicated in matrix-dependent regulation of cell adhesion and motility [19–21]. Furthermore, expression of several additional factors related to cytoskeletal function, such as *WASL* (*N-WASP*), *CFL2*, *RAC1* and *ROCK2*, is reduced following miR-142-3p overexpression. As a consequence of targeting these molecules, miR-142-3p reduces the formation of membrane protrusions and pronounced changes in cell shape and size, ultimately resulting in a significant inhibition of breast cancer cell invasiveness. Our data suggest a novel fundamental posttranscriptional mechanism of cell motility regulation by miR-142-3p, with translational implications for breast cancer pathogenesis and therapy.

## Materials and Methods

### Materials

Media and fetal calf serum supplies were from PAA (Pasching, Germany). Tissue culture supplied from Gibco BRL (Karlsruhe, Germany). Unless stated otherwise, all chemicals were from Sigma (Deisenhofen, Germany).

### Cell culture and microRNA transfection

The human breast cancer cell lines MDA-MB-231, MDA-MB-468 and MCF-7 were purchased from ATCC / LGC Standards (Wesel, Germany) [22]. Cell line authenticity was confirmed by STR analysis. Cells were grown to 70% confluency in 6-well-plates and transiently transfected using Dharmafect reagent (Dharmacon, Lafayette, CO, USA) and 10 nM negative control miRNA #1, miR-142-3p precursor, or 20 nM antimiR-142-3p (all from ABI, Darmstadt, Germany), respectively [8,9]. Functional analyses were performed 72h after transfection. Normal human skin fibroblasts have been previously described [23].

### RNA isolation and reverse transcription

microRNA and mRNA isolation from cultured cells were both performed using the innuPREP RNA Mini Kit (Analytik Jena AG, Jena, Germany) following the manufacturer’s instructions. MicroRNA was converted into cDNA using the TaqMan MicroRNA Reverse Transkription kit (Applied Biosystems Inc., Foster City, USA). Reverse transcription of mRNA was performed using the First strand cDNA Synthesis kit (Fermentas GmbH, St Leon Rot, Germany).

### Cell proliferation assay

MTT (3-(4,5-dimethylthiazol-2-yl)-2,5-diphenyltetrazolium bromide) assay was carried out for 24 h as previously described [9,10]. 72 h after microRNA precursor transfection, 10,000 MDA-MB-231, MDA-MB-468 and MCF-7 cells were seeded in 96-well plates and cultured for 24 h, followed by 24 h incubation in the presence of MTT, lysis and optical density measurement at 595 nm in a microplate reader.

### Cell size measurement

The two-dimensional size [μm^2^] of transfected MDA-MB-231 and MDA-MB-468 cells was measured using phase contrast microscopy and Zeiss AxioVision 4.3 (Zeiss, Jena, Germany) imaging software.

### Cell volume and dry mass determination with digital holographic microscopy (DHM)

Digital off-axis holograms of suspended transfected MDA-MB-468 cells and control cells in DMEM with an osmolality of 320 mOsmol/kg were recorded with a previously described setup [24]. The cell volume (V) was determined from numerically reconstructed quantitative digital holographic microscopy phase images of selected suspended single cells with spherical appearance as previously reported [24,25]. The dry mass was determined as previously described [26].

### Quantitative TaqMan real-time PCR analysis

The indicated cell lines were transfected with a control miR precursor, a miR-142-3p precursor followed by mRNA isolation, cDNA preparation and qPCR (quantitative real-time polymerase chain reaction) [9, 10]. All TaqMan probes were purchased from Applied Biosystems, normalizing to 18S rRNA expression. Data were calculated and expressed as fold change vs control transfected cells using the 2^-ΔΔCt^ method.

### Affymetrix transcriptomic analysis

RNA concentration and purity were measured using a Nanodrop spectrophotometer (Thermo Fisher Scientific Inc., Waltham, MA, USA). RNA integrity was checked with an Agilent Bioanalyzer (software version B.02.07.SI532) on a RNA Nano Chip (both Agilent Technologies Inc., Santa Clara, CA, USA). Affymetrix GeneChip Human Gene 1.0 ST microarrays (Affymetrix UK Ltd., High Wycombe, UK) were performed using the manufacturers´ protocols. The samples were amplified from 100 ng of total RNA in accordance with the Ambion WT Expression assay kit (LifeTechnologies GmbH, Darmstadt, Germany) and fragmented and end-labeled in accordance with the Affymetrix GeneChip WT Terminal Labeling protocol. The prepared targets were hybridized overnight to Affymetrix GeneChip Human Gene 1.0 ST arrays. Following hybridization, the arrays were washed and stained using the Affymetrix GeneChip Fluidics Station 450 and scanned using the Affymetrix GeneChip Scanner 3000 7G. Statistical analyses of microarray data were performed using the Partek Genomics Suite software (Partek Inc., St. Louis, MO, USA). CEL-files (containing raw expression measurements) were imported to Partek GS. The robust multi-array average (RMA) algorithm was used for normalization. The array data were quantile normalized and log-2 transformed. For each probe, a one-way analysis of variance (ANOVA) was performed: **Y**_**ij**_ = **μ** *+* **Type**_**i**_ *+* **Ɛ**_**ij**_, where Y_ij_ represents the j^th^ observation on the i^th^ Type. μ is the common effect for the whole experiment and ε_ij_ represents the random error present in the j^th^ observation on the i^th^ Type. The errors Ɛ_ij_ are assumed to be normally and independently distributed with mean 0 and standard deviation δ for all measurements. For each probe, a student ***t***-test was applied to statistically compare the difference between the means of the groups´ “1423” and “chl” expression measurements. A ***p***-value ≤0.05 was used as a threshold for significance. These data are available through the Gene Expression Omnibus (GEO), accession number GSE50829.

### Luciferase assays

MDA-MB-231, MDA-MB-468 and MCF-7 breast cancer cells were transfected with plasmid pEZX-MT01-N-WASP-3’UTR expressing firefly luciferase (hLuc) under the control of an SV40 enhancer and the 3’UTR of human *ITGAV*, and renilla luciferase (hRLuc) under constitutive control of the cytomegalovirus (CMV) promoter (HmiT055211, GeneCopoeia). An analogous construct was used to investigate regulation of *WASL* (HmiT021768, GeneCopoeia, Rockville, MD, USA). Cells were co-transfected with a control miR precursor or pre-miR-142-3p and simultaneously assayed for activity of both luciferases 72h after transfection [9,10].

### Western Blotting

Extracts of cells transfected with control precursor miR, miR-142-3p or anti-miR-142-3p were analyzed by western-blotting using specific rabbit antibodies against integrin αV, N-WASP (Cell Signaling, Frankfurt a.M., Germany, 1:1,000) and appropriate peroxidase-labeled secondary antibodies (Calbiochem, Darmstadt, Germany, 1:1,000) as previously described [9,10]. Stripped blot membranes were reprobed with mouse-anti-tubulin antibodies (Sigma, Munich, Germany, 1:4,000) and appropriate secondary antibodies as a loading control (N = 3). In addition, extracts of non-transfected MDA-MB-468, MDA-MB-231 and MCF-7 cells were analyzed by western-blotting using mouse mAb anti–β1 integrin (BD Biosciences), mouse mAb anti–β3 integrin (BD Biosciences), and mouse mAb anti-β5 integrin (Santa Cruz), as primary antibodies at the dilutions recommended by the manufacturer (1:500–1:1,000).

### Nano-texture analysis with Atomic Force Microscopy (AFM)

AFM measurements were performed as described before [27]. Briefly, cells were chemically stabilized by 1% glutardialdehyde fixation. AFM was carried out in PBS-buffer using soft, gold-coated cantilevers (0.01 N/m, MLCT, VEECO, Mannheim, Germany) in contact mode at forces less than 5 nN. For texture analysis, subcellular scan areas of (10μm^2^) are recorded. Topographical data of the cell surfaces were analysed using the nAnostic™-method applying customized, proprietary algorithms (Serend-ip GmbH, Münster, Germany) [28]. Nanostructures protruding from the mean surface level are morphometrically evaluated through computer vision. Then, objects are filtered by size and shape; here, only structures of positive local deviational volume (LDV), smaller than 500 nm in height and an area smaller than 1μm^2^ were considered. Number values are extracted for the count of classified objects (per image) and the sum of their deviational volumes (LDVs).

### Confocal immunofluorescence microscopy

MDA-MB-468 and MCF-7 breast cancer cells were subjected to control precursor or miR-142-3p precursor transfection and analyzed by confocal immunofluorescence microscopy on a Leica TCS microscope (Leica, Wetzlar, Germany), or a Zeiss LSM 780 (Carl Zeiss, Jena, Germany) (Z-stacks) using Plan-Apochromat 63x/1.40 Oil DIC objectives. Human skin fibroblasts [23] were used as a control. Samples were prepared as previously described [10] using ALEXA555-phalloidin (Invitrogen, Eugene, OR, USA, 1:1000) for staining of actin filaments, as well rabbit-anti-Integrin-αV (Cell Signaling, 1:100), mouse mAb anti–human integrin αvβ3 (Millipore, 1:100), mouse Ab anti-human vinculin (Sigma, 1:300) and appropriate ALEXA488 or ALEXA594-labeled secondary antibodies (Invitrogen, 1:600). Cell nuclei were visualized by 4’,6’-diamidino-2-phenylindole (DAPI) staining (Sigma, 1:5,000).

### Matrigel invasion assay

24 h after pre-miR transfection, twenty-five thousand cells in 0.5 ml DMEM/10% FCS (fetal calf serum) were added in duplicates to the upper compartments of BioCoat Matrigel Invasion Chambers (BD Biosciences, Heidelberg, Germany). After 24 h, the medium was replaced by serum-free DMEM. To the lower compartment, 0.75 ml DMEM/10% FCS was added. After 48 h, the cells on the lower surface were fixed and stained with Diff-Quik dye (Medion, Düdingen, Switzerland). Excised and mounted filter membranes were photographed using a Zeiss Axiovert microscope equipped with Axiovision software (Zeiss, Jena, Germany) at x100 magnification. For each membrane, cells in five visual fields were counted. Relative invasiveness was expressed as percentage of the cell number on compound-treated inserts compared with control inserts (n>3).

### siRNA-mediated depletion of ITGAV and WASL

Cells were transfected with 20 nM *ITGAV*- or *WASL*-specific siRNA, or a nonspecific control siRNA (all from Ambion, Darmstadt, Germany), essentially as previously described [10]. Knockdown efficiency was confirmed by qPCR and Western blotting. Matrigel invasion chamber assays, quantitative AFM analysis and digital holographic microscopy were performed 72h after transfection.

### Statistics

All experiments were repeated at least three times in duplicates. Data are presented as the mean values±s.e.m. The data were tested for significance employing Student’s unpaired t-test. The level of significance was set at P<0.05.

## Results

### miR-142-3p inhibits invasiveness of human breast cancer cells

**Fig 1:**
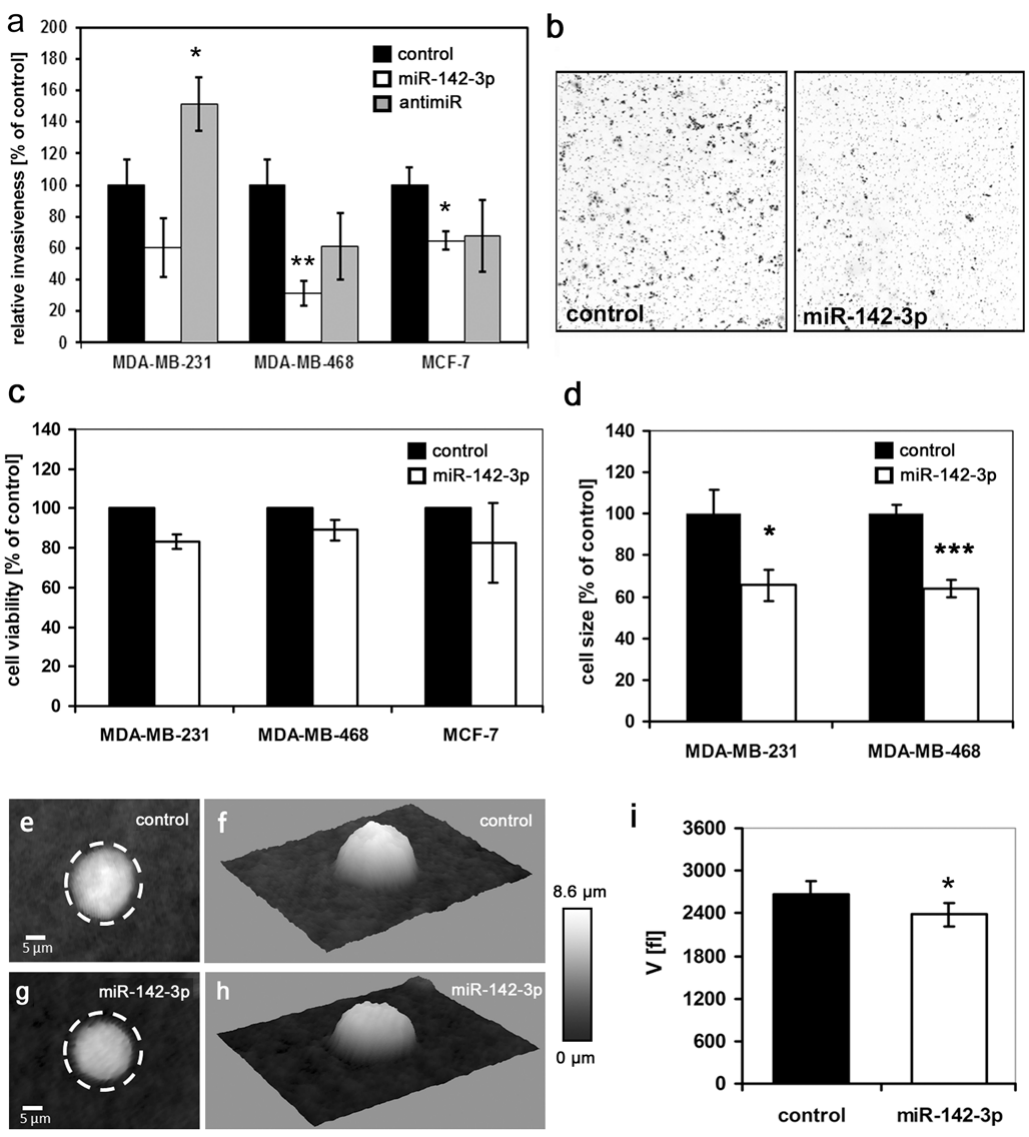
Functional analysis of miR-142-3p upregulation on invasiveness, viability and size of MDA-MB-231, MDA-MB-468 and MCF-7 breast cancer cells. Cells were transfected with a control miRNA, miR-142-3p or antimiR-142-3p as described in the methods section. (a,b) Matrigel invasion assay (BD Biosciences, Heidelberg, Germany) was performed as previously described [9,10]. miR-142-3p significantly inhibited breast cancer cell invasiveness by 39% and 32% in MDA-MB-468 (24h) and MCF-7 cells (48h) respectively. In MDA-MB-231 cells, anti-miR-142-3p caused a significant increase in invasiveness of more than 50%. Error bars = s.e.m (n≥6, *P<0.05, ***P<0.01). (b) Right panel: representative micrographs of invasion filter membranes after crystal violet staining (MDA-MB-468). (c) MTT assay reveals no significant effect of miR-142-3p on cell viability. MDA-MB-231, MDA-MB-468 and MCF-7 cells transfected with miR-142-3p precursors or a control miR precursor were allowed to proliferate for 72h. Error bars = s.e.m., n≥3. (d-i) miR-142-3p upregulation reduces cancer cell diameter and volume. (d) The two-dimensional size [μm^3^] of transfected MDA-MB-231 and MDA-MB-468 cells revealed a significant decrease in size upon miR-142-3p upregulation. N>70, error bars = SEM, *P<0.05, ***P<0.01. (e-i) Digital holographic microscopy (DHM) of miRNA transfected MDA-MB-468 cells reveals decreased cell volume after miR-142-3p transfection. (e,g). Representative quantitative DHM phase contrast images of suspended transfected MDA-MB-468 cells (g) and control cells (e) (coded to 256 gray levels); the dashed circles illustrate the cell volume increase. (f, h) pseudo-three-dimensional representations of the phase images in e, g correspond to the projection of the cell thickness. (i) cell volume V of n = 251 miR-142-3p transfected cells appears significantly decreased in comparison to the volume of n = 172 control cells; Data are mean ± SEM, *P<0.05, ***P<0.001.

In previous studies, miR-142-3p expression correlated with the invasiveness of different breast cancer subtypes [8], with highly invasive bone metastatic MDA-MB-231 cells [29] and with biopsies of the triple-negative subtype [11]. Moreover, miR-142-3p has been identified as part of a signature of six miRNAs which discriminates between tumors from BRCA1/2 mutation carriers and noncarriers with >90% accuracy [30]. We hypothesized that upregulation or inhibition of miR-142-3p expression in breast cancer cells may regulate target molecules playing key roles in breast cancer pathogenesis, thus inducing a more benign phenotype. Using transient microRNA precursor and antimiR inhibitor transfection, we studied the impact of miR-142-3p on invasiveness and cell viability in a panel of three well-established breast cancer cell lines exhibiting different degrees of dedifferentiation, different hormone receptor status, *BRCA1* variant expression levels [31] and malignant potential [22, 32]. This way, we wanted to reflect the heterogeneity of the disease, and investigate if the impact of miR-142-3p on breast cancer cell behaviour is uniform, or restricted to certain subtypes. qPCR analysis in the cell lines MDA-MD-231 (mesenchymal morphology, estrogen receptor negative (ER–), claudin-low, highly invasive and tumorigenic in nude mice), MDA-MB-468 (weakly luminal epithelial-like, ER–, Ki67 high, intermediate invasiveness, forms tumors in nude mice) and MCF-7 (luminal-epithelial-like, ER+, Ki67 low, weakly invasive *in vitro*), revealed about 80-fold higher miR-142-3p expression in MDA-MB-231 cells compared to MDA-MB-468 and MCF-7, which exhibited similar expression levels (S1 Fig). For functional studies, cells were transiently transfected with a control miRNA precursor, pre-miR-142-3p, or an antimiR-142-3p, respectively. Successful transfection was confirmed by qPCR (S1 Fig). In MDA-MB-468 and MCF-7 cells, miR-142-3p upregulation inhibited *in vitro* Matrigel invasiveness by 68% and 35%, respectively (Fig 1A and 1B). While miR-142-3p upregulation yielded highly variable results in MDA-MB-231 cells, anti-miR-142-3p transfection increased invasiveness by more than 50% (Fig 1A). miR-142-3p upregulation had no significant impact on cell viability (Fig 1C). However, phase contrast and digital holographic microscopy revealed a significant decrease in cell size and volume induced by miR-142-3p (Fig 1D–1I), suggesting that changes in cell shape and size may be linked to the invasion phenotype.

### miR-142-3p upregulation reduces expression of gene products linked to cytoskeletal function and cell motility

**Fig 2:**
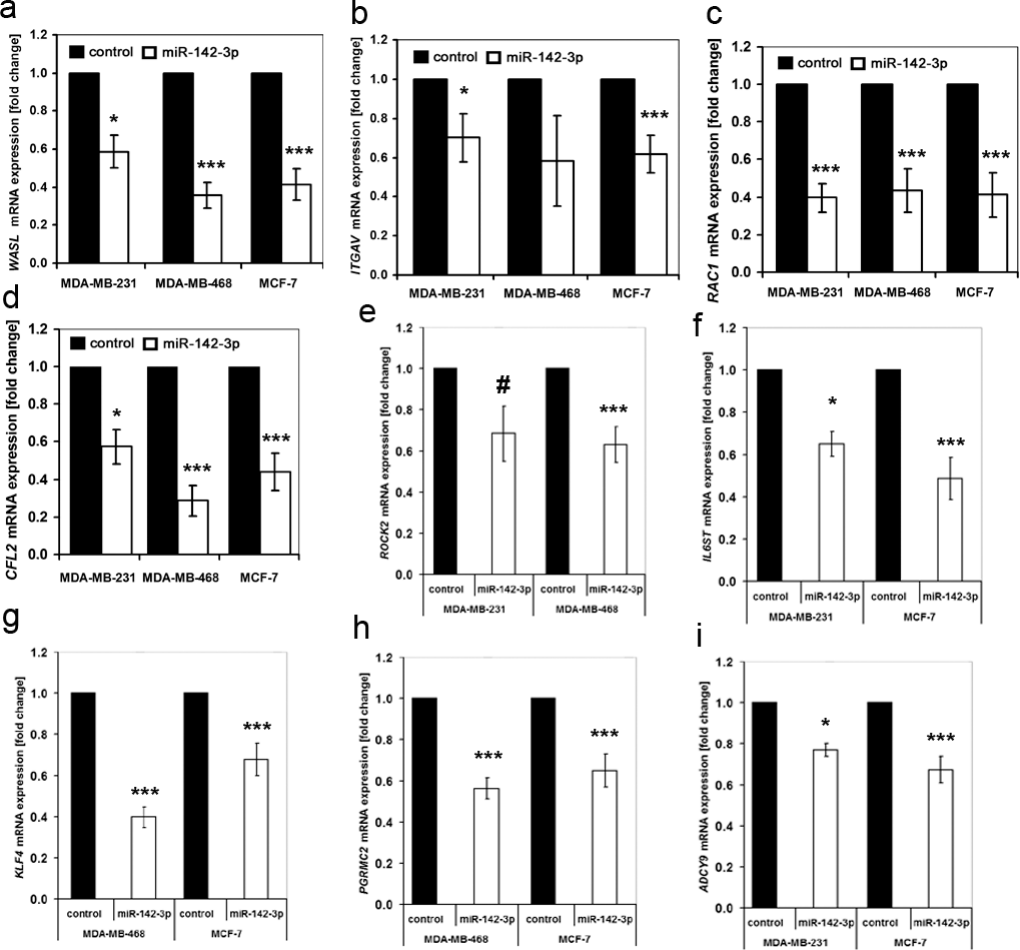
Confirmation of differentially regulated mRNA targets in miR-142-3p vs control transfected breast cancer cell lines by qPCR. Cells were transfected with a control miRNA, miR-142-3p or antimiR-142-3p as described in the methods section, followed by RNA and cDNA preparation and qPCR analysis. (a-i). Black columns = control, white columns = miR-142-3p precursor, N>6, error bars = SEM, #P = 0.052 (n.s.), *P<0.05, ***P<0.001. miR-142-3p upregulation inhibits expression of *WASL* (a), *ITGAV* (MDA-MB-231; MCF-7) (b), *RAC1* (c), *CFL2* (d), *ROCK2* (MDA-MB-231 with P = 0.052, MDA-MB-468) (e), *IL6ST* (MDA-MB-231, MCF-7) (f), *KLF4* (MDA-MB-468, MCF-7) (g), *PGRMC2* (MDA-MB-468, MCF-7) (h), and *ADCY9* (MDA-MB-468, MCF-7) (i).

*In silico* analysis using the MiRanda-algorithm [33] yielded more than 4300 predicted mRNA targets of miR-142-3p. To screen for miR-142-3p-regulated candidate genes, we compared the transcriptome of miR-142-3p-overexpressing MDA-MB-231 cells against that of control miRNA precursor-transfected cells using Affymetrix U133v2 Gene Arrays. 76 downregulated and 132 upregulated genes fulfilled the filtering criteria of P<0.05 and fold change ≥1.5 (S1 Table). Considering their gene ontology groupings, several proteins were potentially linked to the observed changes in invasiveness: The genes encoding Integrin-αV, a transmembrane protein involved in breast cancer metastasis and proliferation [19–21], N-WASP, a protein involved in signaling from cell membrane receptors to the actin cytoskeleton [34–36], Rac1, a cell motility-regulating Rho-GTPase involved in cancerogenesis [16,37] and Cofilin-2, a protein modulating actin reorganization and thus cell motility [38] were downregulated upon pre-miR-142-3p transfection, as confirmed by qPCR (Fig 2A–2D). Downregulation of additional predicted miR-142-3p targets was confirmed in two of the three cell lines (Fig 2E–2I). These mRNAs included the Rho-GTPase-associated kinase *ROCK2 [10,39],* the interleukin-6 receptor subunit *IL6ST [40],* the pluripotency-associated transcription factor *KLF4* [41], progesterone membrane component *PGRMC2* [42], and the adenylate cyclase *ADCY9*, an inflammation-related, known target of miR-142-3p [13]. With the exception of an upregulation of *ROCK2* in MDA-MB-231 and of *FLT1* in MCF-7 cells (S2 Fig), antimiR-treatment did not significantly affect expression of all investigated predicted [33] miR-142-3p targets (results not shown).

### ITGAV and WASL are direct targets of post-transcriptional regulation by miR-142-3p

**Fig 3:**
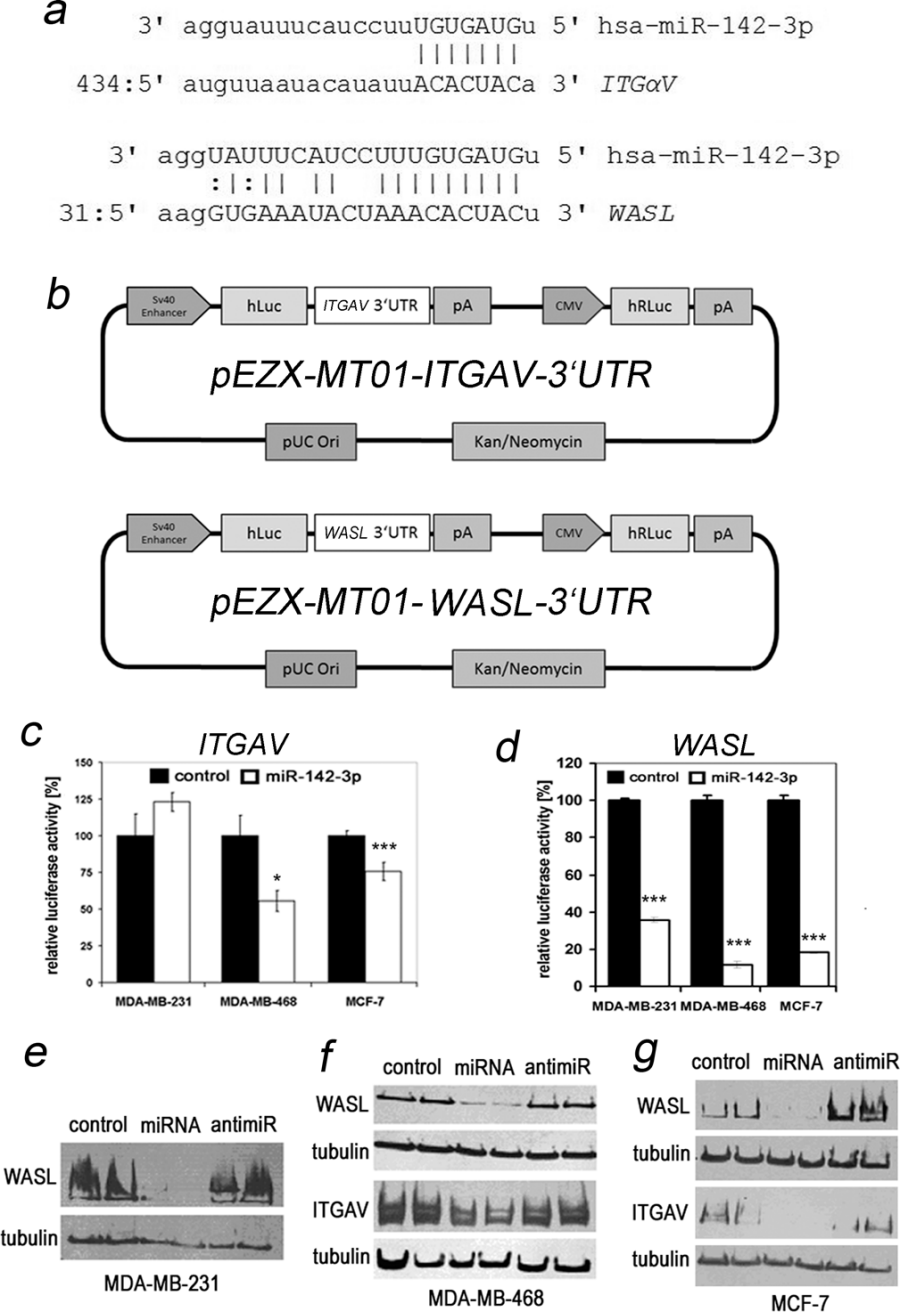
3’UTR luciferase assay and Western blotting identify Integrin alpha V and N-WASP as relevant regulatory targets of miR-142-3p. (a) Alignment of the seed sequence of miR-142-3p with predicted target sites of the 3’UTR of *ITGAV* (top) and one of the three predicted target sites of *WASL* (bottom) according to the microRNA.org database [28]. (b,c) The 3’UTR of *ITGAV* is a target for transcriptional regulation by miR-142-3p. Cells were transfected with plasmid pEZX-MT01-*ITGAV*-3’UTR (b, upper panel) expressing firefly luciferase (hLuc) under the control of an SV40 enhancer and the 3’UTR of human *ITGAV*, and renilla luciferase (hRLuc) under constitutive control of the cytomegalovirus (CMV) promoter. Cells were cotransfected with a control pre-miR or pre-miR-142-3p and simultaneously assayed for activity of both luciferases 72h after transfection. (b) pA = poly-A tail, pUCOri = origin of replication, Kan/NeomycinR = antibiotic resistance genes. (c) miR-142-3p transfection induced a significant decrease in *ITGAV*-specific normalized firefly luciferase activity in MCF-7 and MDA-MB-468, but not in MDA-MB-231 cells (N = 3, *P<0.05, ***P<0.001). (d) The 3’UTR of *WASL* is a target for transcriptional regulation by miR-142-3p Cells were transfected with plasmid pEZX-MT01-*WASL*-3’UTR (b, lower panel). Cells were cotransfected with a control pre-miR or pre-miR-142-3p and simultaneously assayed for activity of both luciferases 72h after transfection. *WASL*-dependent luciferase activity is reduced ranging from 64% to 88% in all three cell lines (N = 3, ***P<0.001). (e-g) Western blot confirmation of differentially regulated Integrin-αV and N-WASP protein expression in miR-142-3p vs. control transfected breast cancer cell lines. miR-142-3p upregulation inhibits N-WASP expression in all investigated cell lines (e-g), which can be reversed by anti-miR inhibition. miR-142-3p precursor transfection inhibits Integrin-αV expression in MDA-MB-468 (f) and MCF-7 (g) cells. The migration position of molecular weight markers relative to Integrin-αV and N-WASP is shown in S4 Fig.

According to the miRanda algorithm provided by the microRNA.org database [33], the 3’UTRs of *ITGAV* and *WASL* are predicted to be targeted by miR-142-2p (Fig 3A). To formally prove direct regulation of the *ITGAV* 3’UTR by miR-142-3p, we performed luciferase assays employing a plasmid allowing for simultaneous constitutive expression of renilla luciferase, and of firefly luciferase under the control of the 3’UTR of human *ITGAV* (Fig 3B upper panel). pre-miR-142-3p transfection of MDA-MB-468 and MCF-7 cells resulted in significant decreases of in normalized firefly luciferase activity (Fig 3C). Using an analogous *WASL* 3’UTR-assay (Fig 3B lower panel), a significant regulation could be confirmed for all three cell lines (Fig 3D). miR-142-3p-dependent downregulation of Integrin-αV (MCF-7, MDA-MB-468) and N-WASP (all cell lines) was further confirmed at the protein level through Western Blotting (Fig 3E–3G; S3 Fig). Although Rac1 is targeted by miR-142-3p in hepatocellular carcinoma cells [16], we could not confirm altered expression at the protein level (results not shown). Cycloheximide inhibitor studies revealed that Rac1 protein levels were stable within our timeframe of investigation in the absence of new protein synthesis (K. Brüggemann, unpublished data).

### Transfection with pre-miR-142-3p causes loss of membrane protrusions in MDA-MB-468 cells

**Fig 4:**
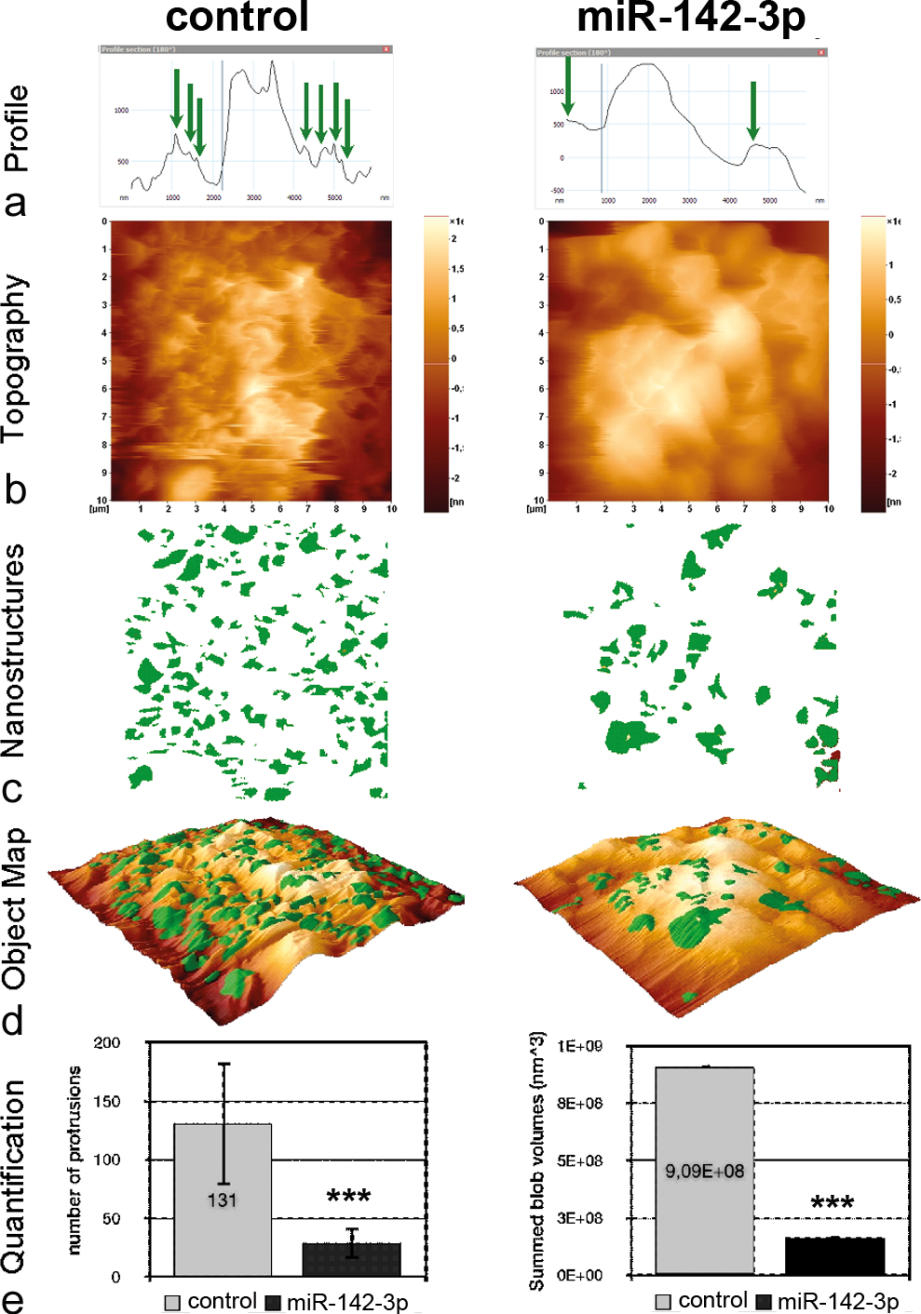
Nano-texture analysis. MDA-MB-468 cell surfaces without (left column) or with treatment by miR-142-3p (right column) were imaged by Atomic Force Microscopy (AFM) at nanometer resolution. Shown are (a) representative height profiles, (b) topography raw data of a (10 μm)^2^ scan, (c) protruding structure elements (green), (d) the overlay of (a) and (b) in a 3D-map and (e) the number values for the object count and the total volume (given as the sum of individual sizes (LDVs, local deviational volumes) per image). Shown are the mean values ± SD per image for n = 5, ***P<0.001.

As miR-142-3p-dependent regulation of cytoskeletal structures may be involved in the observed invasion phenotype, we preformed further morphological investigations using atomic force microscopy (AFM). Typical height profiles obtained by Nano Texture Analysis demonstrate a smoother surface of cells treated with miR-142-3p as compared to the fine ruffles exhibited by control cells (Fig 4, arrows). This observation is supported from mere inspection of the raw topographic images. When selecting spike-like protrusions by computer vision, it is obvious that their number is substantially diminished by miR-142-3p (green spots). Accordingly, the mean height of protrusions is reduced from 365 nm to 228 nm (data not shown). The distribution of objects does not exhibit a specific pattern; the location on the cell topography can be seen in the 3D-representation (Fig 4). Importantly, the object number is significantly reduced from 153 (control) to 61 (miRNA treated) and the sum of object volumes is significantly reduced accordingly from 1.29 fl to 0.49 fl (fl = 10–15 l) (Fig 4).

### miR-142-3p overexpression results in substantially altered cell morphology

**Fig 5:**
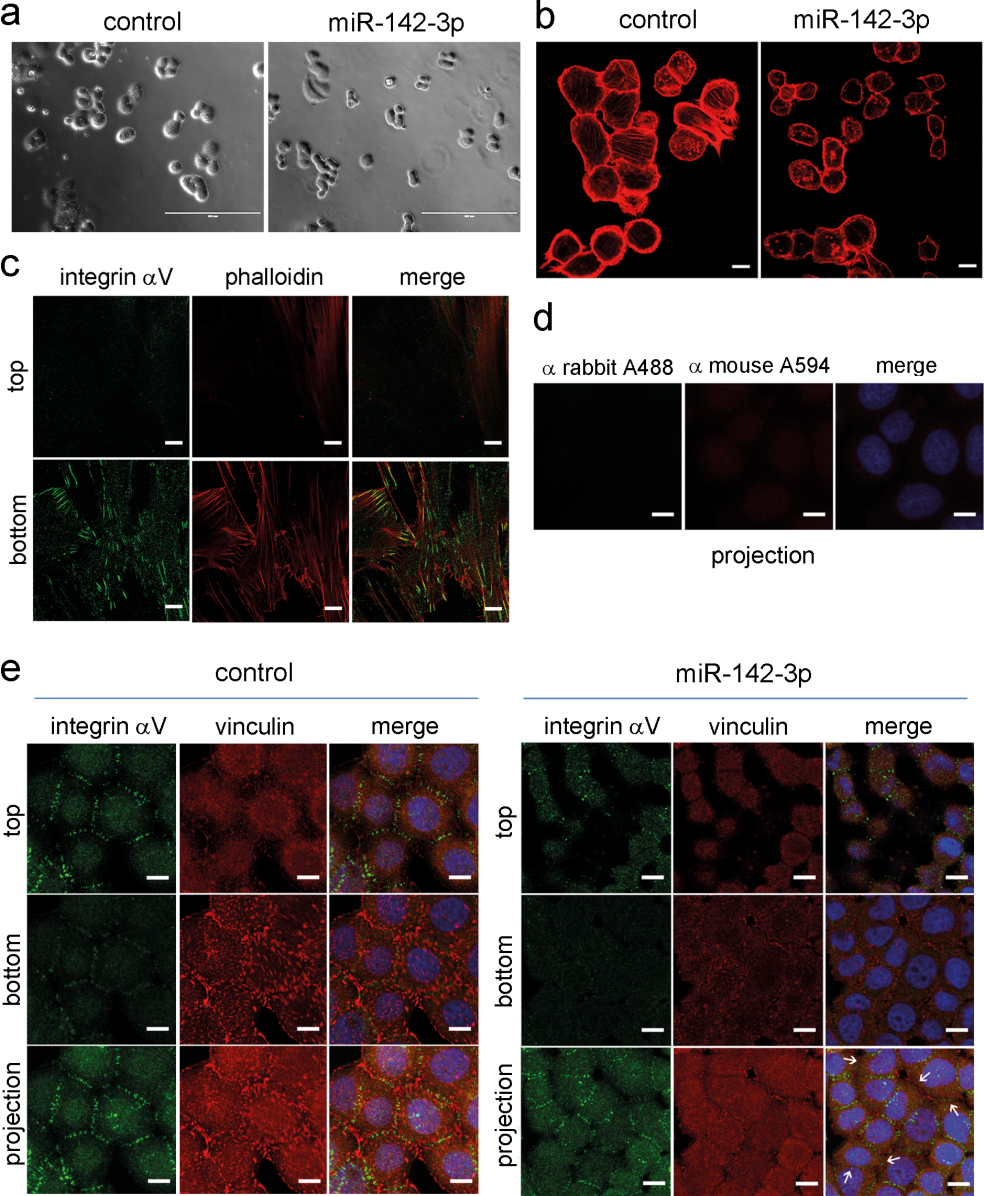
miR-142-3p induces a change in cellular morphology. MDA-MB-468 breast cancer cells were subjected to control precursor or miR-142-3p precursor transfection, and analyzed by phase contrast (a) and confocal immunofluorescence microscopy (b-e). Human skin fibroblasts were analysed for control purposes (c). Shown are phalloidin staining of actin filaments (red), staining of vinculin (red) and staining of Integrin-αV (green). Yellow colour denotes colocalization in merged images, whereas blue colour indicates DAPI nuclear staining in merged panels (d) and (e). (a) phase contrast image shows reduced size of miR-142-3p transfected cells compared to controls (scale bar = 200 μm). (b) miR-142-3p induces a change in cell morphology towards a more rounded shape with fewer membrane protrusions and less actin stress fibers. (see Fig 4). Confocal microscopy analyis of Z-stacks reveals localization of integrin-αV in a focal adhesion-like pattern in human skin fibroblasts (c), whereas a staining in cell-cell-contacts is observed in MDA-MB-468 cells (e). Shown are the top and bottom focal planes and projections. Use of secondary antibodies alone did not generate a signal in MDA-MB-468 cells (d). Compared to controls, miR-142-3p-transfected MDA-MB-468 cells showed a reduced accumulation of integrin-αV in cell-cell contacts (arrows). Scale bar in panels b-e = 10μm.

**Fig 6:**
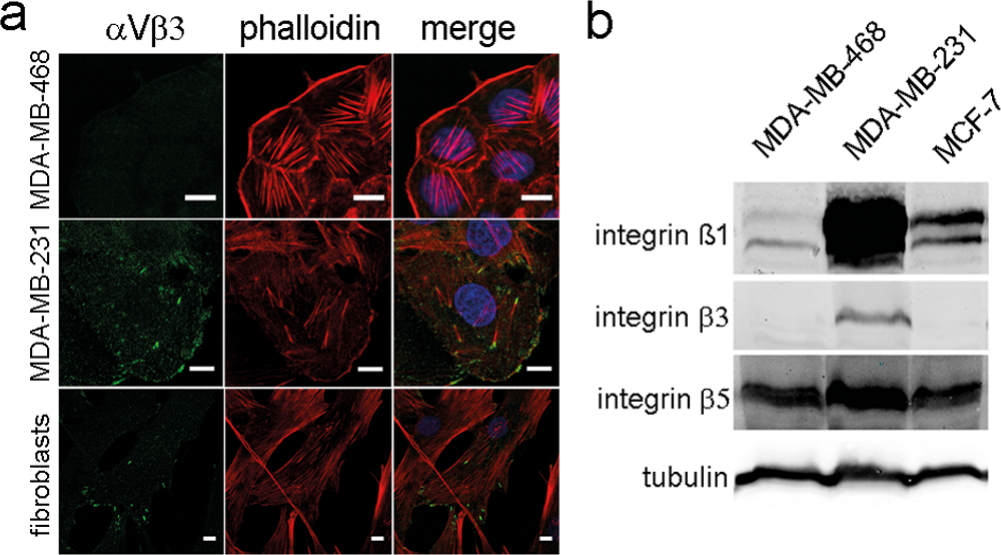
Expression of β-integrin subunits in human breast cancer cell lines. The expression of, αVβ3-, β1-, β3-, and β5- integrin in MDA-MB-468, MDA-MB-231 and MCF-7 cells was studied by confocal immunofluorescence (αVβ3) (a) or western blotting(β1-, β3-, β5-) (b), respectively, as described in Figs 3 and 5. Only MDA-MB-231 cells showed expression of (αV)β3-integrin, in partial colocalization with actin stress fibers in a fibroblast-like pattern. All breast cancer cell lines studied expressed β1-, and β5- integrin subunits (b), albeit in different quantities.

**Fig 7:**
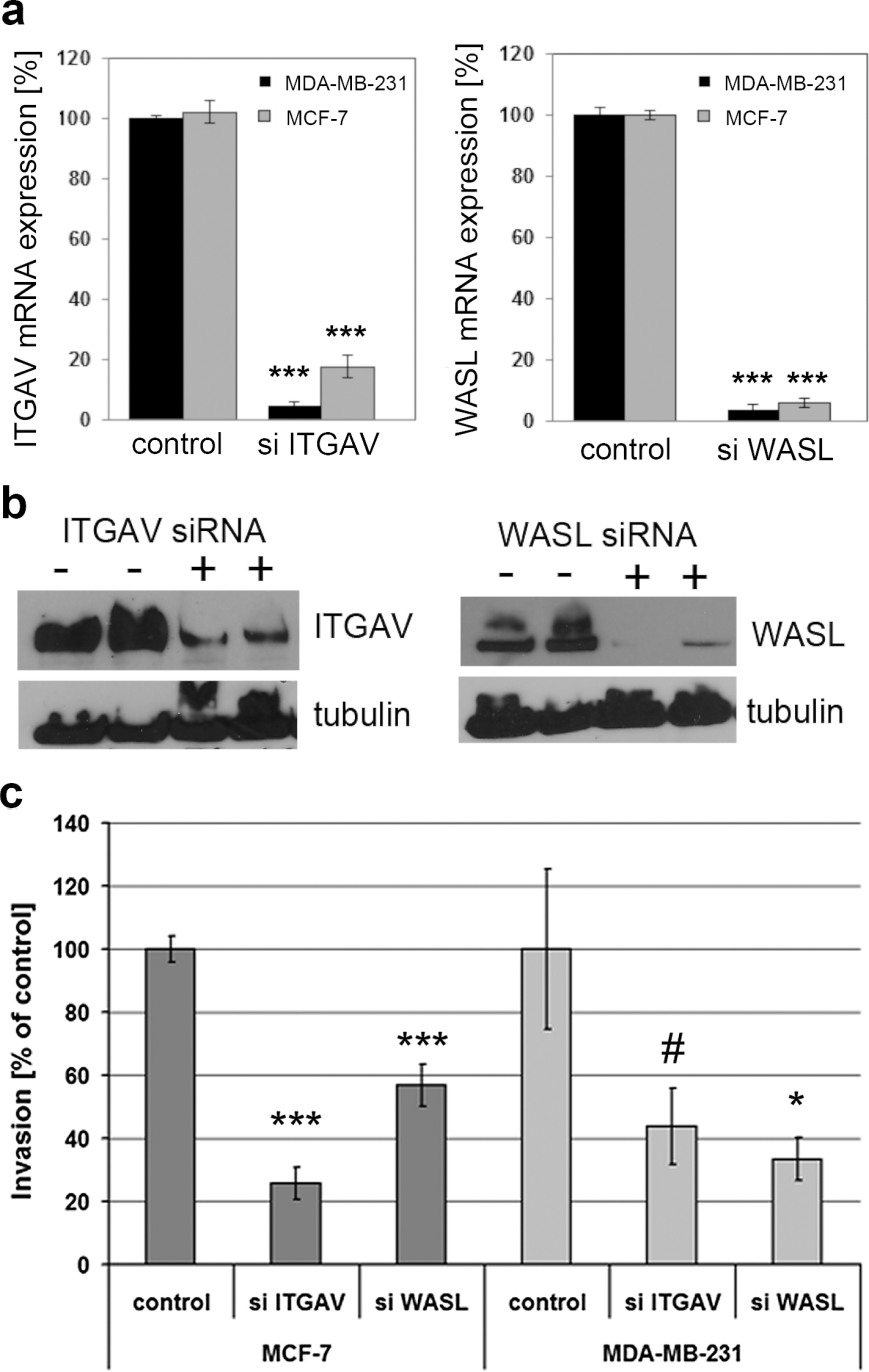
siRNA-mediated depletion of *WASL* or *ITGAV* reduces matrigel invasiveness of MCF-7 and MDA-MB-231 cells. (a) Confirmation of successful siRNA knockdown of *ITGAV* (left panel) and *WASL* (right panel) by qPCR. N = 4, ***P<0.001. (b) Confirmation of successful siRNA knockdown of integrin-αV and WASL at the protein level by Western blotting. See Fig 3E–3G for details. (c) ITGAV and WASL-depletion reduces matrigel invasiveness of MDA-MB-231 and MCF-7 cells. N>5, #P = 0.06 (n.s.), *P<0.05, ***P<0.001.

**Fig 8:**
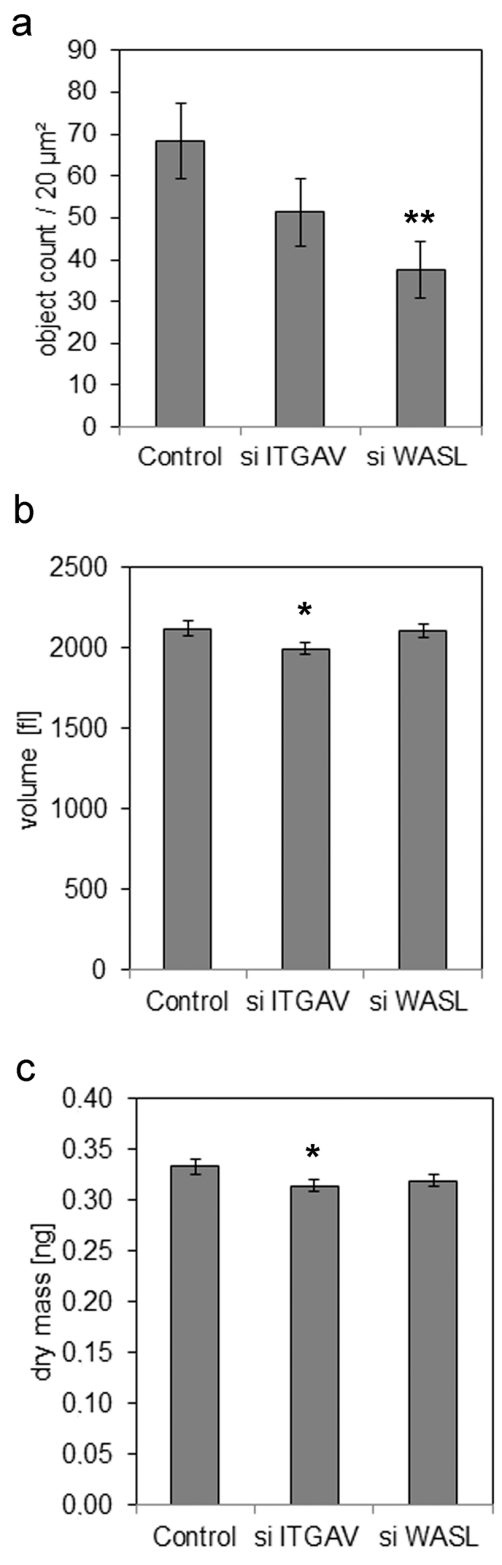
siRNA-mediated depletion of *WASL* or *ITGAV* differentially affect membrane protrusions, cell volume and dry mass of MDA-MB-468 cells. MDA-MB-468 cells were subjected to control, *ITGAV* or *WASL* siRNA treatment, followed by analysis via AFM (a) or DHM (b,c), 72h after transfection. (a) MDA-MB-468 cell surfaces were imaged by Atomic Force Microscopy (AFM) at nanometer resolution. Quantitative analysis of object counts as a readout of membrane protrusion formation reveals a significant reduction in cells treated with *WASL* siRNA (** = p<0.01, n = 15, error bars = s.e.m.). (b,c) Digital holographic microscopy of siRNA transfected MDA-MB-468 cells reveals decreased cell volume (b) and dry mass (c) after *ITGAV* siRNA transfection compared to control siRNA-transfected cells. *P<0.05, N = 200 cells per group, data are mean ± SEM.

We next performed confocal immunofluorescence microscopy to analyze cell morphology using a complementary approach. Phase contrast microscopy and staining of the actin cytoskeleton with fluorescently labelled phalloidin confirmed a reduction of cell size (Fig 5A and 5B, S4 Fig). Formation of actin stress fibers was less evident in miR-142-3p-transfected cells compared to controls, and actin appeared to have a more cortical distribution. N-WASP staining confirmed reduced expression in pre-miR-142-3p-transfected cells but did not reveal altered subcellular localization (S5 Fig). However, in control siRNA-treated MDA-MB-468 cells, immunofluorescent staining and analysis of Z-stacks revealed a prominent localization of integrin-αV at intercellular junctions, and little or no colocalization with the focal adhesion protein vinculin (Fig 5E). Integrin-αV expression was reduced following miR-142-3p transfection. Notably, while most sites of cell-cell contact were positive for integrin-αV in control cells, several neighbouring miR-142-3p transfected cells did not show integrin-αV staining (Fig 5E, arrows), resulting in a fusiform morphology. The intercellular integrin staining was not due to aberrant reactivity, as confirmed by secondary antibody negative controls (Fig 5D), and a proper focal adhesion-like staining pattern in human skin fibroblasts (Fig 5C). We next analyzed the expression of β-integrin subunits in MDA-MB-468, MDA-MB-231 and MCF-7 cells. Immunofluorescence microscopy and Western blot analysis revealed that integrin-αVβ3 was only expressed in MDA-MB-231 cells and fibroblasts, but not in MDA-MB-468 or MCF-7 cells (Fig 6A and 6B). In contrast to the intercellular integrin-αV staining observed in MDA-MB-468 cells (Fig 5E), integrin-αVβ3 showed a focal-adhesion-like staining pattern in MDA-MB-231 cells (Fig 6A). Western blot analysis furthermore confirmed that all cell lines expressed integrin-β5 and integrin-β1, as reported previously [43]. While tubulin-normalized integrin-β5 levels were comparable between cell lines, integrin-β1 was strongly expressed by MDA-MB-231 cells, followed by intermediate levels in MCF-7, and rather weak expression in MDA-MB-468 cells (Fig 6B). We next aimed at identifying the individual contributions of *WASL* and *ITGAV* to the miR-142-3p dependent cell phenotype, employing siRNA technology. *WASL* depletion by siRNA significantly inhibited MCF-7 and MDA-MB-231 invasiveness, whereas *ITGAV* knockdown resulted in significant inhibition of MCF-7 Matrigel invasion and a trend for inhibition in MDA-MB-231 cells (P = 0.06, N = 8) (Fig 7), thus generating a phenocopy of the miR-142-3p-mediated impact on cellular invasiveness. Quantitative morphological analysis of MDA-MB-468 cells subjected to *ITGAV-* and *WASL*-siRNA knockdown revaled a significantly reduced number of membrane protrusions upon *WASL*-depletion, as determined by AFM (Fig 8A), suggesting a major contribution of *WASL* to this miR-142-3p-induced phenotype. In contrast, only *ITGAV*-, but not *WASL*-siRNA knockdown, induced a significant reduction of cell volume and dry mass (Fig 8B and 8C).

## Discussion

In this functional in vitro study, we have demonstrated a novel role for miR-142-3p, a miRNA dysregulated in breast cancer compared to healthy tissue [8,11] in the regulation of breast cancer cell invasiveness. Within the cell lines investigated in this study, miR-142-3p showed a non-uniform distribution, which is in accordance with a differential expression of miR-142-3p in different subtypes of breast cancer [8,11]. The relatively higher expression of miR-142-3p in MDA-MB-231 cells compared to MCF-7 and MDA-MB-468 may be the reason for the efficiency of antimiR-inhibition in these cells, whereas MCF-7 and MD-MB-468 already displayed low miR-142-3p expression, and were thus less prone to the inhibitory approach. Using advanced microscopy techniques, we demonstrated that reduced invasiveness is associated with reduced numbers of membrane protrusions, alterations in cell shape and reductions in cancer cell size and volume. While Affymetrix analysis of the transciptome of miR-142-3p overexpressing cells revealed a dysregulation of numerous mRNAs compared to controls (S1 Table), a group of cytoskeletal elements and regulators of cytoskeletal function has emerged as functionally relevant prime candidates responsible for the phenotypic changes. Among these, WASL, an Arp2/3-dependent regulator of actin polymerization [34–36], was identified as a major contributor to the antiinvasive action of miR-142-3p. Not only was its 3’UTR-dependent regulation by miR-142-3p confirmed at the mRNA and protein levels, its siRNA depletion also generated a phenocopy of the invasion phenotype, and substantially reduced the formation of membrane protrusions. Indeed, reduction of *WASL* expression renders macrophages rounder and less polarized, in accordance with the morphological changes observed in our breast cancer cells [44]. The authors could furthermore demonstrate that podosomes of *WASL*-depleted macrophages were unable to degrade extracellular matrix due to a mistargeting of metalloproteinases, thus providing a link to an invasion phenotype. The *WASL*-dependent formation of invasive membrane protrusions has been identified as an important mechanism of triggering invasive behaviour of human breast cancer cells, and can be induced by several pathways including estrogen- [45], PDGF- [46] and EGF-induced signaling [47]. According to our results, it would be conceivable that miR-142-3p-dependent regulation of *WASL* is involved in the generation of microspikes, since it is responsible for the branching of f-actin strands [34,36,48], and a developing branch will go through a stage of spiking upwards. An inhibition of branching will therefore diminish the protrusions spiking out of the cell. Indeed, protrusions essential for in vivo migration and invasion of tumor cells are formed dependent on WASL [49,50]. Notably, WASL function is closely related to integrins [51], and several signal transducers downstream of this family of heterodimeric matrix receptors, including the kinases FAK [45] and ROCK [52], and small GTPases of the Rho family [53,54].

Notably, the integrin subunit *ITGAV* was identified as a regulatory target for miR-142-3p in MCF-7 and MDA-MB-468 cells. Consistent with the antiinvasive effect of *ITGAV* siRNA knockdown in our study, a role of *ITGAV* in modulating breast cancer metastasis was previously demonstrated, identifying a matrix-dependent role of this integrin in breast cancer cell adhesion and migration [55,56], and a functional interplay with p38 MAPK and uPA [57] as mechanistic aspects. Indeed, drugs blocking *ITGAV* function have been successful in preclinical mouse models of breast cancer metastasis [20,21], underscoring the importance of miR-142-3p-dependent *ITGAV* regulation in our in vitro system. Surprisingly, we could not demonstrate regulation of *ITGAV* by miR-142-3p in MDA-MB-231 cells at the 3’UTR and protein level, although this mode of regulation was utilized in MDA-MB-468 and MCF-7 cells. We can only speculate on the underlying mechanisms. It is conceivable that a miR-142-3p-dependent factor responsible for posttranscriptional regulation of *ITGAV* is expressed in MDA-MB-231 cells, but not in the other cell lines, thus enabling to neutralize the effect seen in MDA-MB-468 and MCF-7 cells. Indeed, a differential regulation of some of the miRNA targets identified in this study exists and may have modulated the cellular response (Fig 2), as *ROCK2* is not significantly regulated by miR-142-3p in MCF-7 cells, *ADCY9* and *IL6ST* are not regulated in MDA-MB-468 cells, whereas *KLF4* and *PGRMC2* are not regulated in MDA-MB-231 cells. Moreover, crosstalk between different integrin subunits is known to affect their mRNA stability [58], and differential integrin expression patterns in our cell lines may have affected the experimental results. Indeed, an analysis of β-integrin subunit expression in our cell lines revealed that the β3-subunit was only expressed in MDA-MB-231 cells, but not in MCF-7 and MDA-MB-468 cells. In combination with integrin-αV, different β-integrin subunits can affect cancer cell behavior via different mechanisms. For example, αvβ1 induces the proinvasive process of epithelial-to-mesenchymal transition via binding to tenascin C [59], whereas β3-subunits affect this process by modulating TGFβ-signaling [60]. In contrast, integrin αVβ5 promotes breast cancer cell invasiveness in a PAK4-dependent mechanism [61]. Independent of the β-subunit involvement, a reduced expression of integrin-αV would be expected to result in reduced invasiveness of the affected breast cancer cell line, which is consistent with our findings. With respect to differentially affected cell behaviour, it has to be considered that MDA-MB-231 cells harbor numerous activating mutations, which lead to context dependent responses to growth factor stimulation [62] and miRNAs [9,63]. For example, miR-145 is not capable of modulating MDA-MB-231 proliferation and apoptosis because of a TP53 mutation and an ER-negative status, whereas it is capable of doing so in TP53 wild-type cells [63]. Possibly, similar mechanisms could play in the role in the case of miR-142-3p and *ITGAV* in MDA-MB-231 cells. While a reduction in invasiveness by miR-142-3p upregulation was seen in all three cell lines, this effect was not statistically significant in MDA-MB-231 cells, indicating a more variable response that may be linked to the higher basal expression level of miR-142-3p compared to the other cell lines (S1 Fig). Therefore, a further upregulation of miR-142-3p may have lower impact in these cells compared to MDA-MB-468 and MCF-7 cells expressing lower levels, whereas antimiR-treatment had the opposite effect. Nevertheless, the increased invasiveness of MDA-MB-231 cells treated with antimiR-142-3p would be in line with the observed inhibitory effect of *WASL* siRNA depletion on MDA-MB-231 cells. Impairment of the actin cytoskeleton-associated function of integrin-αVβ3 [64] may have additionally contributed to the impact of *ITGAV*-siRNA depletion in MDA-MB-231 cells. However MDA-MB-468 apparently utilize a different mechanism, as integrin-αV was localized at cell-cell contacts. Indeed, there is evidence that αV-integrins can localize to cell-cell junctions [65,66]. Importantly, in pancreatic and breast carcinoma, αvβ3 integrin forms a complex with c-Src kinase, and this complex is signalling active in the absence of ligand occupation, i.e. when cells are grown in suspension [67]. These findings thus indicate that αv integrins can have a signalling function in the absence of ligand binding. Our siRNA studies on *ITGAV* indicate that this function is linked to the invasion phenotype, and to the regulation of cell size, which may be linked to cell rounding upon loss of cell-cell contact and reduced activation of signaling pathways downstream of integrin-αV [68].

Due to their mode of action, which is based both on fully and partially complementary base pairing of the miRNAs seed sequence with cognate mRNAs, miRNAs are capable of modulating the expression of more than one target [3,4]. This concept is confirmed by the results of our transcriptomic analysis, which showed a differential expression of 208 mRNAs upon miR-142-3p upregulation in MDA-MB-231 cells (S1 Table). *IL6ST, KLF4 and PGRMC2* have been linked to cancer pathogenesis [40–42], and downregulation of their mRNA expression by miR-142-3p may therefore be worth elucidating in future studies. Similarly, the upregulation of the protease inhibitors *SERPINB2* and *SERPINA6* in miR-142-3p-transfected cells (S1 Table) may have contributed to the antiinvasive phenotype. With respect to factors modulating cytoskeletal function, a downregulation of Rac1, Cofilin-2 and *ROCK2* may have additionally influenced the invasion phenotype. Indeed, the small GTPase Rac1 affects cell spreading, directed cell migration and lamellipodia formation, and has been identified as a regulatory target of miR-142-3p in hepatocellular carcinoma cells [16]. A recent study could demonstrate that overexpression of constitutively active Rac1 is capable of suppressing a spreading defect of MDA-MB-231 cells caused by miR-149-dependent targeting of Rap1a and Rap1b [37], demonstrating the importance of this cytoskeletal regulator for cell adhesion and motility. Likewise, cofilin-2 is an important regulator of actin dynamics [69], and has been shown to be overexpressed in agressive forms of breast cancer [38]. While long-term downregulation of *RAC1* and *CFL2* mRNA levels by miR-142-3p in patient tumor tissues may be of relevance for metastatic spread, it cannot serve as an explanation for our *in vitro* observations, as a downregulation at the protein level was not observed due to a high protein stability, or low turnover rates, respectively (K. Brüggemann, unpublished).

In summary, this study has revealed a novel role for miR-142-3p in regulating breast cancer cell invasiveness, which could at least partially be attributed to a targeting of *WASL* and *ITGAV*, and possibly additional cytoskeletal regulators. Various modes of therapeutic miRNA delivery via microvesicle-mediated intravenous application, nanocomplexes, viral vectors, non-viral carriers or local application are currently under investigation and have been successfully applied in preclinical models for a variety of miRNAs [70–72]. Therefore, future investigations towards a role of miR-142-3p as a potential anti-metastasis drug for breast cancer appear rewarding in order to expand our understanding of miR-142-3p as a novel cytoskeletal regulator beyond basic cell biology.

## Supporting Information

S1 Fig Characterization of miR-142-3p expression in human breast cancer cell lines. a) Basal miR-142-3p expression levels relative to MDA-MB-468. microRNA was isolated from the indicated breast cancer cell lines, reverse transcribed and analyzed by TaqMan qPCR using ABI probes exactly as described in Götte et al. (2010). RNU6B served as a housekeeping RNA control. N = 3, error bars = SD. b) qPCR confirmation of successful upregulation of miR-142-3p after transfection with miR-142-3p and control miRNA precursors (see main manuscript and Götte et al. (2010) for details). microRNA was isolated 72h after transfection. N>3, error bars = SEM.
(PPT)

S2 Fig Significant upregulation of target genes by antimiR-142-3p-mediated inhibition in human breast cancer cell lines. qPCR using ABI TaqMan probes for *ROCK2* (MDA-MB-231) or *FLT1* (MCF-7) expression normalized to 18S rRNA expression after transfection with antimiR-142-3p and control miRNA precursor (see main manuscript and Götte et al. (2010) for details). N = 8, error bars = SEM, *P<0.05.
(PPT)

S3 Fig Migration position of N-WASP and Integrin-αV in Western blot analysis. Western blotting analysis of lysates of MDA-MB-468 cells subjected to miR-142-3p upregulation (miR-142-3p) and inhibition (anti-miR-142-3p) was performed as decribed in Fig 3F of the main manuscript. Mr indicates the migration position of molecular weight markers (Thermo Scientific PAGE ruler).
(PPT)

S4 Fig miR-142-3p induces a change in cell morphology and actin cytoskeleton structure in MCF-7 cells. Following transfection with a negative control miRNA, miR-142-3p precursors (all from ABI), cells were processed for immunohistochemistry as described in the main manuscript using ALEXA555-phalloidin (Invitrogen, Eugene, OR, USA, 1:1,000) for staining of actin filaments. miR-142-3p transfection induces a more rounded cell morphology and a more cortical actin distribution.
(PPT)

S5 Fig miR-142-3p modulates expression levels, but not subcellular distribution of N-WASP in MDA-MB-468 cells. Following transfection with a negative control miRNA, miR-142-3p precursors or an antimiR-142-3p (all from ABI), cells were processed for immunohistochemistry as described in the main manuscript using rabbit-anti-N-WASP (Cell signaling, 1:100) and appropriate ALEXA488-labeled secondary antibodies (Invitrogen, 1:600). N-WASP localizes to the cell periphery and to regions of cell-cell contact.
(PPT)

S1 Table Transcriptional changes (<1.5-fold, p<0.05) in miR-142-3p-transfected compared to control miRNA-transfected MDA-MB-231 cells according to Affymetrix screening on three biological replicates. The GEO accession number of this screening is GSE50829. See text for details.
(DOC)
