# Supplementary material for: microRNA miR-142-3p Inhibits Breast Cancer Cell Invasiveness by Synchronous Targeting of WASL, Integrin Alpha V, and Additional Cytoskeletal Elements
Source: PLoS One. 2015 Dec 10;10(12):e0143993. doi: 10.1371/journal.pone.0143993 (PMC4675527; doi:10.1371/journal.pone.0143993)
Supplement: S1 Fig — a) Basal miR-142-3p expression levels relative to MDA-MB-468. microRNA was isolated from the indicated breast cancer cell lines, reverse transcribed and analyzed by TaqMan qPCR using ABI probes exactly as described in Götte et al. (2010). RNU6B served as a housekeeping RNA control. N = 3, error bars = SD. b) qPCR confirmation of successful upregulation of miR-142-3p after transfection with miR-142-3p and control miRNA precursors (see main manuscript and Götte et al. (2010) for details). microRNA was isolated 72h after transfection. N>3, error bars = SEM. (PPT) [file pone.0143993.s001.ppt]

## Slide 1
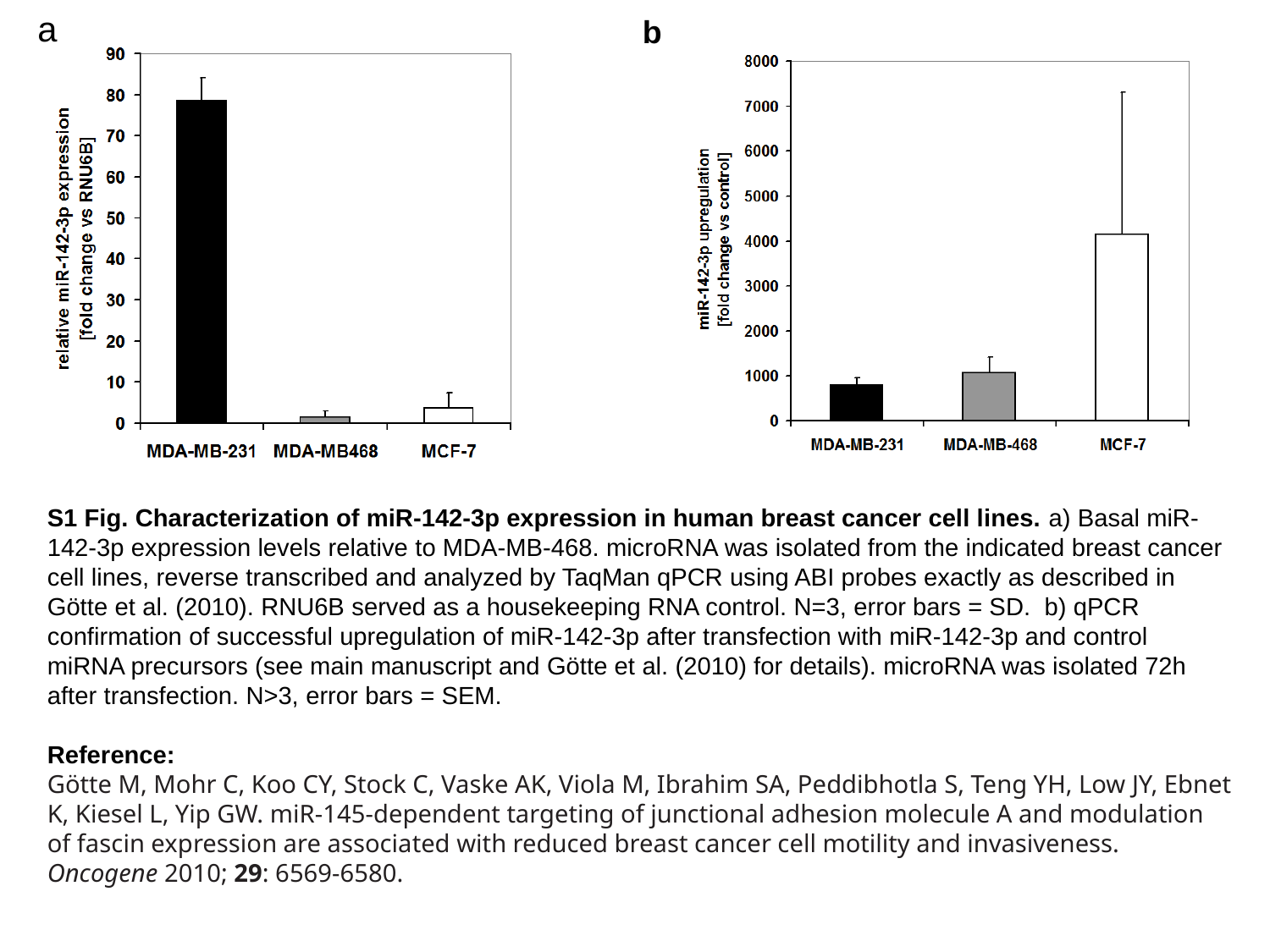

a
b
S1 Fig. Characterization of miR-142-3p expression in human breast cancer cell lines. a) Basal miR-142-3p expression levels relative to MDA-MB-468. microRNA was isolated from the indicated breast cancer cell lines, reverse transcribed and analyzed by TaqMan qPCR using ABI probes exactly as described in Götte et al. (2010). RNU6B served as a housekeeping RNA control. N=3, error bars = SD. b) qPCR confirmation of successful upregulation of miR-142-3p after transfection with miR-142-3p and control miRNA precursors (see main manuscript and Götte et al. (2010) for details). microRNA was isolated 72h after transfection. N>3, error bars = SEM.
Reference:
Götte M, Mohr C, Koo CY, Stock C, Vaske AK, Viola M, Ibrahim SA, Peddibhotla S, Teng YH, Low JY, Ebnet K, Kiesel L, Yip GW. miR-145-dependent targeting of junctional adhesion molecule A and modulation of fascin expression are associated with reduced breast cancer cell motility and invasiveness. Oncogene 2010; 29: 6569-6580.
