# Supplementary material for: microRNA miR-142-3p Inhibits Breast Cancer Cell Invasiveness by Synchronous Targeting of WASL, Integrin Alpha V, and Additional Cytoskeletal Elements
Source: PLoS One. 2015 Dec 10;10(12):e0143993. doi: 10.1371/journal.pone.0143993 (PMC4675527; doi:10.1371/journal.pone.0143993)
Supplement: S2 Fig — qPCR using ABI TaqMan probes for ROCK2 (MDA-MB-231) or FLT1 (MCF-7) expression normalized to 18S rRNA expression after transfection with antimiR-142-3p and control miRNA precursor (see main manuscript and Götte et al. (2010) for details). N = 8, error bars = SEM, *P<0.05. (PPT) [file pone.0143993.s002.ppt]

## Slide 1
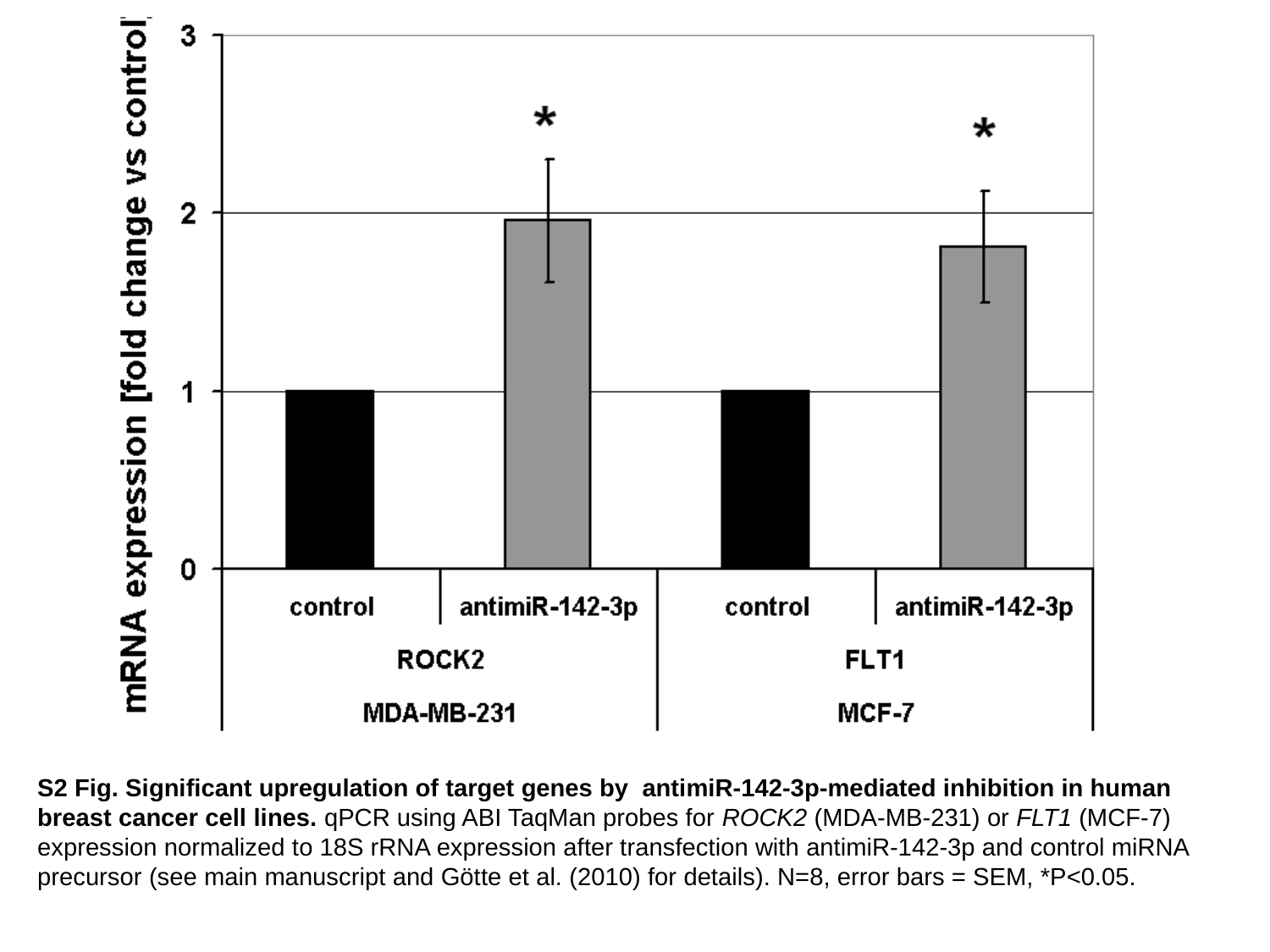

S2 Fig. Significant upregulation of target genes by antimiR-142-3p-mediated inhibition in human breast cancer cell lines. qPCR using ABI TaqMan probes for ROCK2 (MDA-MB-231) or FLT1 (MCF-7) expression normalized to 18S rRNA expression after transfection with antimiR-142-3p and control miRNA precursor (see main manuscript and Götte et al. (2010) for details). N=8, error bars = SEM, *P<0.05.
