# Supplementary material for: microRNA miR-142-3p Inhibits Breast Cancer Cell Invasiveness by Synchronous Targeting of WASL, Integrin Alpha V, and Additional Cytoskeletal Elements
Source: PLoS One. 2015 Dec 10;10(12):e0143993. doi: 10.1371/journal.pone.0143993 (PMC4675527; doi:10.1371/journal.pone.0143993)
Supplement: S3 Fig — Western blotting analysis of lysates of MDA-MB-468 cells subjected to miR-142-3p upregulation (miR-142-3p) and inhibition (anti-miR-142-3p) was performed as decribed in Fig 3F of the main manuscript. Mr indicates the migration position of molecular weight markers (Thermo Scientific PAGE ruler). (PPT) [file pone.0143993.s003.ppt]

## Slide 1
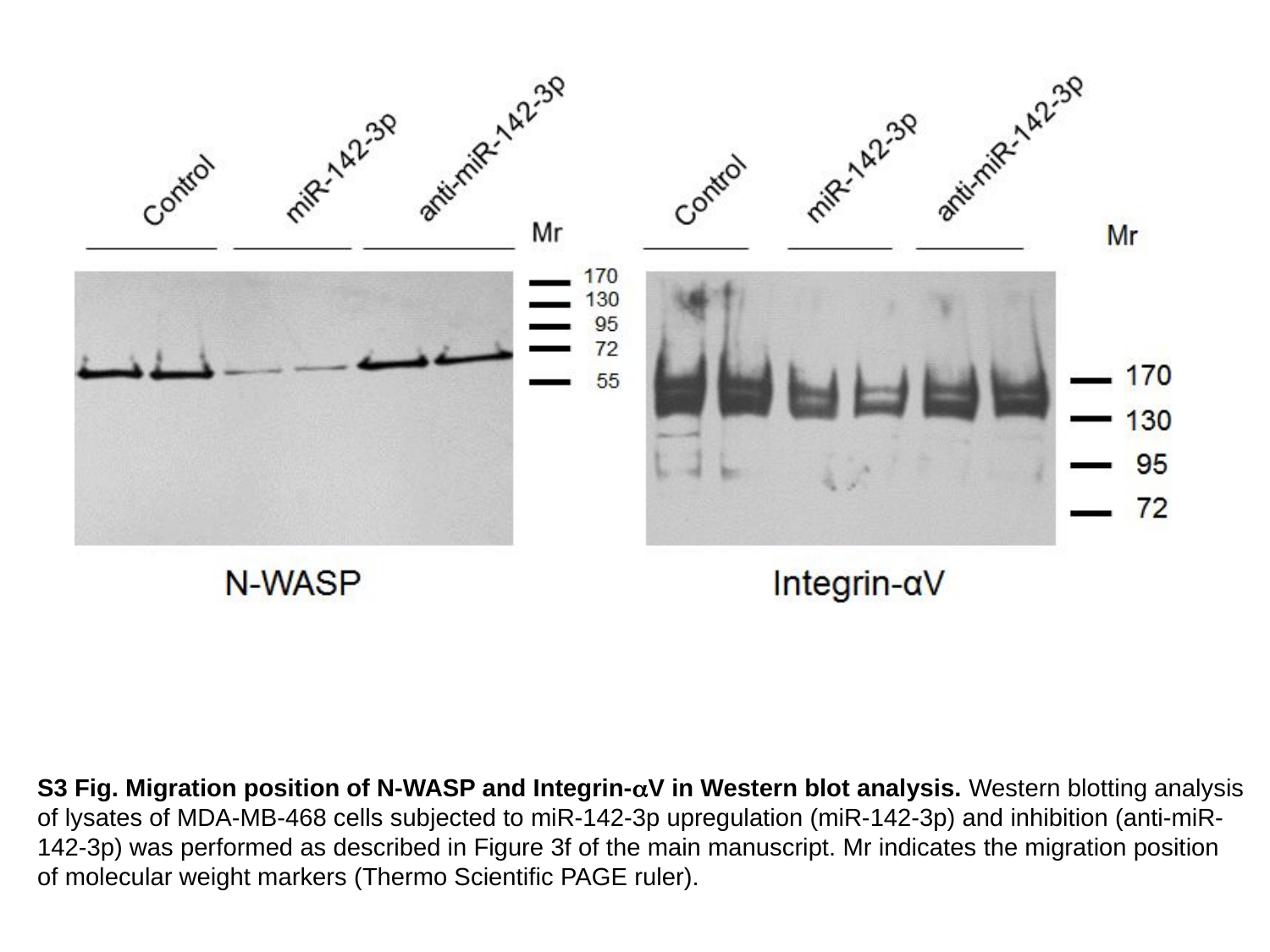

S3 Fig. Migration position of N-WASP and Integrin-αV in Western blot analysis. Western blotting analysis of lysates of MDA-MB-468 cells subjected to miR-142-3p upregulation (miR-142-3p) and inhibition (anti-miR-142-3p) was performed as described in Figure 3f of the main manuscript. Mr indicates the migration position of molecular weight markers (Thermo Scientific PAGE ruler).
