# Supplementary material for: microRNA miR-142-3p Inhibits Breast Cancer Cell Invasiveness by Synchronous Targeting of WASL, Integrin Alpha V, and Additional Cytoskeletal Elements
Source: PLoS One. 2015 Dec 10;10(12):e0143993. doi: 10.1371/journal.pone.0143993 (PMC4675527; doi:10.1371/journal.pone.0143993)
Supplement: S4 Fig — Following transfection with a negative control miRNA, miR-142-3p precursors (all from ABI), cells were processed for immunohistochemistry as described in the main manuscript using ALEXA555-phalloidin (Invitrogen, Eugene, OR, USA, 1:1,000) for staining of actin filaments. miR-142-3p transfection induces a more rounded cell morphology and a more cortical actin distribution. (PPT) [file pone.0143993.s004.ppt]

## Slide 1
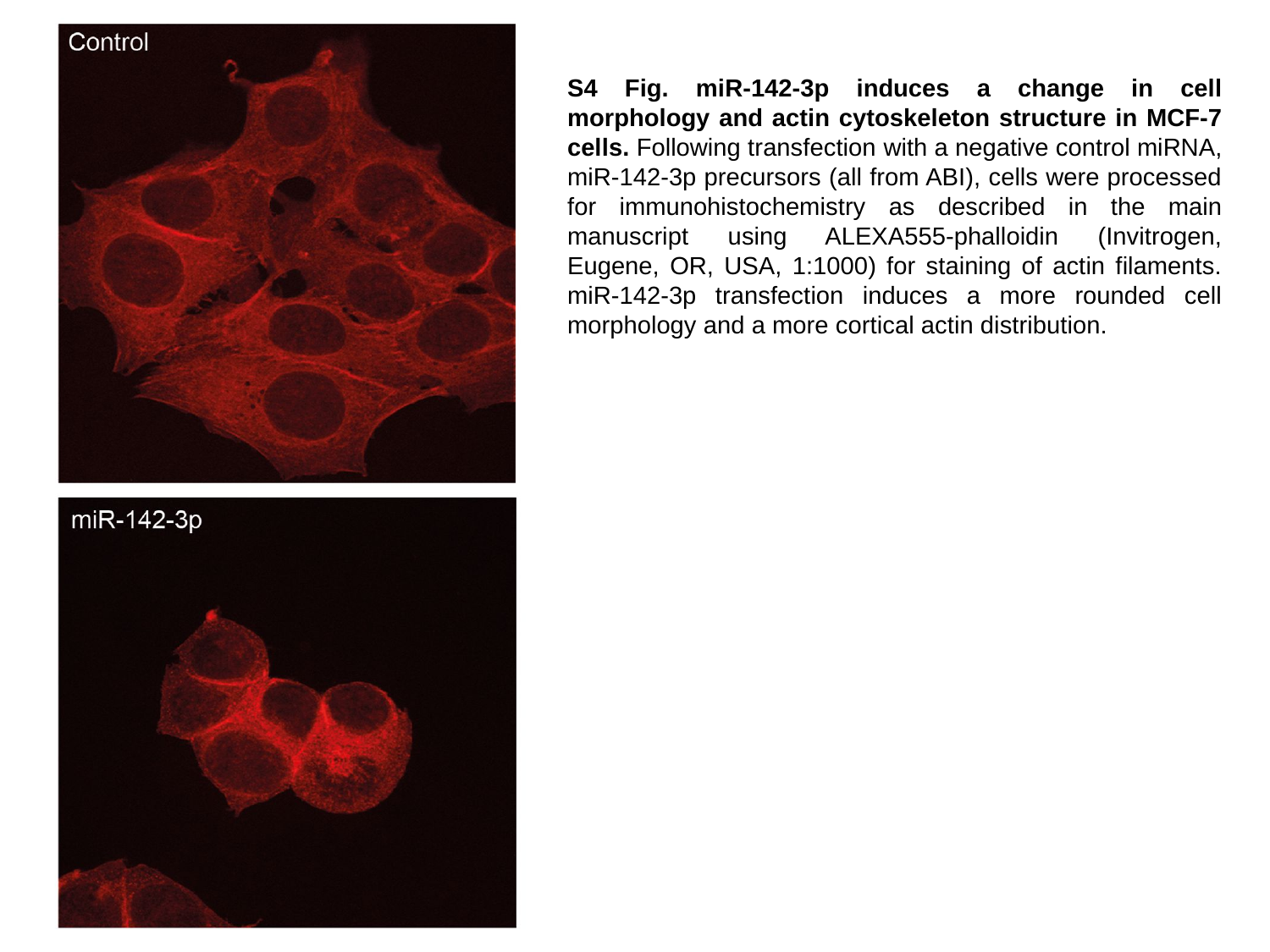

S4 Fig. miR-142-3p induces a change in cell morphology and actin cytoskeleton structure in MCF-7 cells. Following transfection with a negative control miRNA, miR-142-3p precursors (all from ABI), cells were processed for immunohistochemistry as described in the main manuscript using ALEXA555-phalloidin (Invitrogen, Eugene, OR, USA, 1:1000) for staining of actin filaments. miR-142-3p transfection induces a more rounded cell morphology and a more cortical actin distribution.
