# Supplementary material for: microRNA miR-142-3p Inhibits Breast Cancer Cell Invasiveness by Synchronous Targeting of WASL, Integrin Alpha V, and Additional Cytoskeletal Elements
Source: PLoS One. 2015 Dec 10;10(12):e0143993. doi: 10.1371/journal.pone.0143993 (PMC4675527; doi:10.1371/journal.pone.0143993)
Supplement: S5 Fig — Following transfection with a negative control miRNA, miR-142-3p precursors or an antimiR-142-3p (all from ABI), cells were processed for immunohistochemistry as described in the main manuscript using rabbit-anti-N-WASP (Cell signaling, 1:100) and appropriate ALEXA488-labeled secondary antibodies (Invitrogen, 1:600). N-WASP localizes to the cell periphery and to regions of cell-cell contact. (PPT) [file pone.0143993.s005.ppt]

## Slide 1
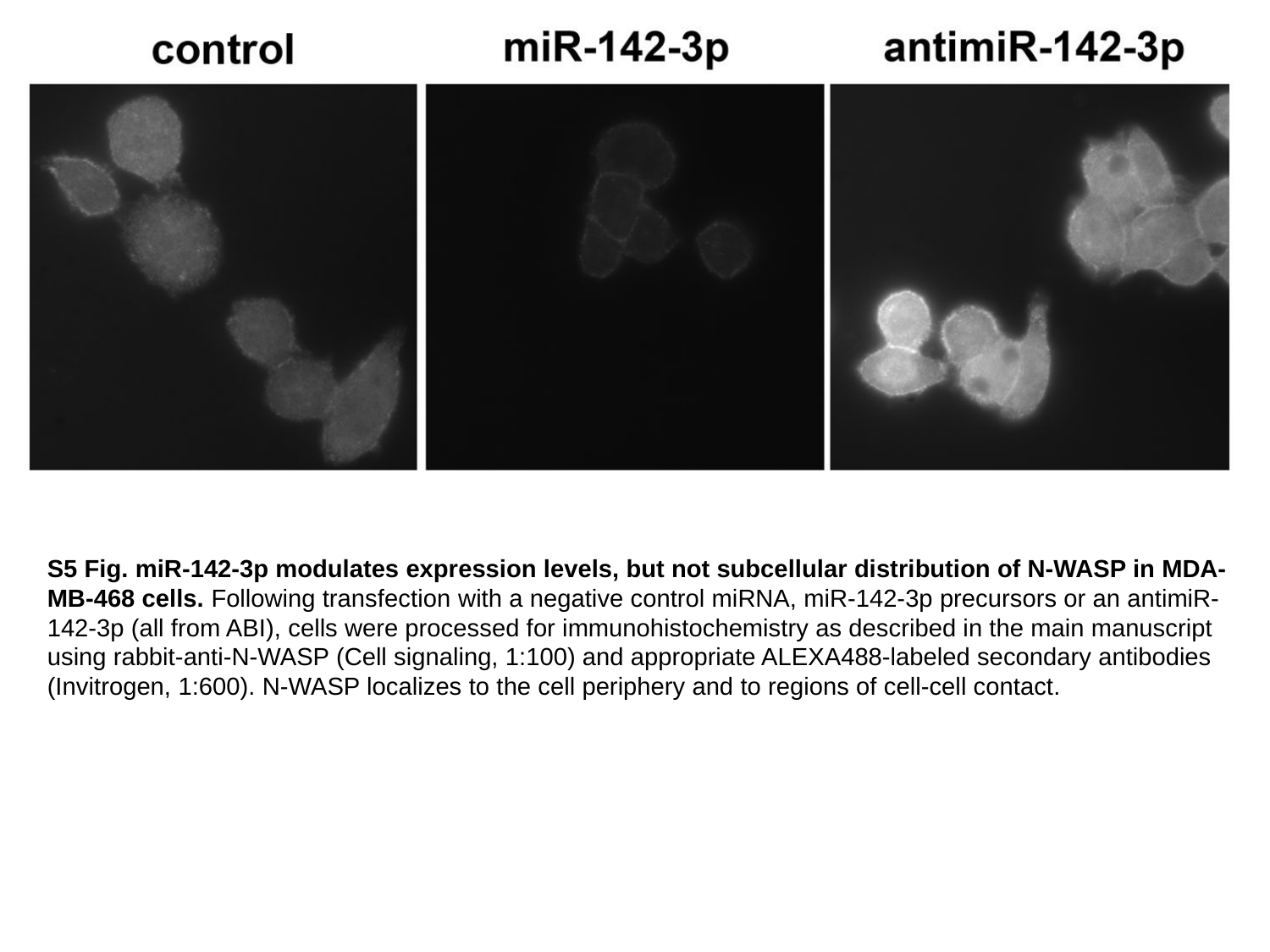

S5 Fig. miR-142-3p modulates expression levels, but not subcellular distribution of N-WASP in MDA-MB-468 cells. Following transfection with a negative control miRNA, miR-142-3p precursors or an antimiR-142-3p (all from ABI), cells were processed for immunohistochemistry as described in the main manuscript using rabbit-anti-N-WASP (Cell signaling, 1:100) and appropriate ALEXA488-labeled secondary antibodies (Invitrogen, 1:600). N-WASP localizes to the cell periphery and to regions of cell-cell contact.
