# Supplementary material for: microRNA miR-142-3p Inhibits Breast Cancer Cell Invasiveness by Synchronous Targeting of WASL, Integrin Alpha V, and Additional Cytoskeletal Elements
Source: PLoS One. 2015 Dec 10;10(12):e0143993. doi: 10.1371/journal.pone.0143993 (PMC4675527; doi:10.1371/journal.pone.0143993)
Supplement: S1 Table — The GEO accession number of this screening is GSE50829. See text for details. (DOC) [file pone.0143993.s006.doc]

**S1 Table. Transcriptional changes (<1.5-fold, p<0.05) in miR-142-3p-transfected compared to control miRNA-transfected MDA-MB-231 cells according to Affymetrix screening on three biological replicates.** The GEO accession number of this screening is GSE50829. See text for details.

| **Gene Symbol** | **Transcript Cluster ID** | **gene_assignment** | **RefSeq** | **Fold-Change** | **Regulation** |
| --- | --- | --- | --- | --- | --- |
| *WASL* | 8142671 | NM_003941 // WASL // Wiskott-Aldrich syndrome-like // 7q31.3 // 8976 /// ENST000 | NM_003941 | -2,97926 | down |
| *BOD1* | 8115865 | NM_138369 // BOD1 // biorientation of chromosomes in cell division 1 // 5q35.2 / | NM_138369 | -2,20463 | down |
| *MANBAL* | 8062371 | NM_022077 // MANBAL // mannosidase, beta A, lysosomal-like // 20q11.23-q12 // 63 | NM_022077 | -2,08638 | down |
| *CFL2* | 7978586 | NM_021914 // CFL2 // cofilin 2 (muscle) // 14q12 // 1073 /// NM_138638 // CFL2 / | NM_021914 | -2,07492 | down |
| *PAEP* | 8159265 | NM_001018049 // PAEP // progestagen-associated endometrial protein // 9q34 // 50 | NM_001018049 | -2,01645 | down |
| *TWF1* | 8013906 | NM_002822 // TWF1 // twinfilin, actin-binding protein, homolog 1 (Drosophila) // | NM_002822 | -1,95476 | down |
| *VAMP3* | 7897370 | NM_004781 // VAMP3 // vesicle-associated membrane protein 3 (cellubrevin) // 1p3 | NM_004781 | -1,87069 | down |
| *SERPINB4* | 8023688 | NM_002974 // SERPINB4 // serpin peptidase inhibitor, clade B (ovalbumin), member | NM_002974 | -1,84912 | down |
| *TWF1* | 7962441 | NM_002822 // TWF1 // twinfilin, actin-binding protein, homolog 1 (Drosophila) // | NM_002822 | -1,84535 | down |
| *AKT1S1* | 8038477 | NM_032375 // AKT1S1 // AKT1 substrate 1 (proline-rich) // 19q13.33 // 84335 /// | NM_032375 | -1,83336 | down |
| *ARL2* | 7941104 | NM_001667 // ARL2 // ADP-ribosylation factor-like 2 // 11q13 // 402 /// ENST0000 | NM_001667 | -1,81331 | down |
| *FAM114A1* | 8094609 | NM_138389 // FAM114A1 // family with sequence similarity 114, member A1 // 4p14 | NM_138389 | -1,81124 | down |
| *FAM127B* | 8175302 | NM_001078172 // FAM127B // family with sequence similarity 127, member B // Xq26 | NM_001078172 | -1,79006 | down |
| *TNFRSF9* | 7912145 | NM_001561 // TNFRSF9 // tumor necrosis factor receptor superfamily, member 9 // | NM_001561 | -1,75623 | down |
| *GAL* | 7942064 | NM_015973 // GAL // galanin prepropeptide // 11q13.3 // 51083 /// ENST0000026564 | NM_015973 | -1,75471 | down |
| *SLC16A6* | 8017843 | NM_001174166 // SLC16A6 // solute carrier family 16, member 6 (monocarboxylic ac | NM_001174166 | -1,75372 | down |
| *FAM73A* | 7902476 | BX537792 // FAM73A // family with sequence similarity 73, member A // 1p31.1 // | BX537792 | -1,75105 | down |
| *YES1* | 8021984 | NM_005433 // YES1 // v-yes-1 Yamaguchi sarcoma viral oncogene homolog 1 // 18p11 | NM_005433 | -1,7414 | down |
| *GPC6* | 7969613 | NM_005708 // GPC6 // glypican 6 // 13q32 // 10082 /// ENST00000377047 // GPC6 // | NM_005708 | -1,73756 | down |
| *C20orf194* | 8064637 | NM_001009984 // C20orf194 // chromosome 20 open reading frame 194 // 20p13 // 25 | NM_001009984 | -1,7078 | down |
| *ZBTB41* | 7923119 | NM_194314 // ZBTB41 // zinc finger and BTB domain containing 41 // 1q31.3 // 360 | NM_194314 | -1,66857 | down |
| *CLDN12* | 8134091 | NM_001185072 // CLDN12 // claudin 12 // 7q21 // 9069 /// NM_001185073 // CLDN12 | NM_001185072 | -1,66841 | down |
| *TXNIP* | 7904726 | NM_006472 // TXNIP // thioredoxin interacting protein // 1q21.1 // 10628 /// ENS | NM_006472 | -1,66628 | down |
| *PMEPA1* | 8067233 | NM_020182 // PMEPA1 // prostate transmembrane protein, androgen induced 1 // 20q | NM_020182 | -1,64569 | down |
| *TSEN34* | 8031145 | NM_024075 // TSEN34 // tRNA splicing endonuclease 34 homolog (S. cerevisiae) // | NM_024075 | -1,64407 | down |
| *FAM83D* | 8062571 | NM_030919 // FAM83D // family with sequence similarity 83, member D // 20q11.22- | NM_030919 | -1,6394 | down |
| *CFB* | 8178115 | NM_001710 // CFB // complement factor B // 6p21.3 // 629 /// ENST00000417261 // | NM_001710 | -1,63914 | down |
| *PAQR5* | 7984524 | NM_001104554 // PAQR5 // progestin and adipoQ receptor family member V // 15q23 | NM_001104554 | -1,63618 | down |
| *INPP5A* | 7931479 | NM_005539 // INPP5A // inositol polyphosphate-5-phosphatase, 40kDa // 10q26.3 // | NM_005539 | -1,63482 | down |
| *CFB* | 8179351 | NM_001710 // CFB // complement factor B // 6p21.3 // 629 /// ENST00000417261 // | NM_001710 | -1,63237 | down |
| *C1S* | 7953603 | NM_201442 // C1S // complement component 1, s subcomponent // 12p13 // 716 /// N | NM_201442 | -1,62432 | down |
| *CFB* | 8118345 | NM_001710 // CFB // complement factor B // 6p21.3 // 629 /// ENST00000425368 // | NM_001710 | -1,62143 | down |
| *GPX3* | 8109333 | NM_002084 // GPX3 // glutathione peroxidase 3 (plasma) // 5q23 // 2878 /// ENST0 | NM_002084 | -1,61934 | down |
| *NUCKS1* | 7909142 | NM_022731 // NUCKS1 // nuclear casein kinase and cyclin-dependent kinase substra | NM_022731 | -1,60718 | down |
| *CDK16* | 8167103 | NM_006201 // CDK16 // cyclin-dependent kinase 16 // Xp11 // 5127 /// NM_033018 / | NM_006201 | -1,60696 | down |
| *MNS1* | 7989146 | NM_018365 // MNS1 // meiosis-specific nuclear structural 1 // 15q21.3 // 55329 / | NM_018365 | -1,60337 | down |
| *CNIH4* | 7910014 | NM_014184 // CNIH4 // cornichon homolog 4 (Drosophila) // 1q42.11 // 29097 /// E | NM_014184 | -1,59888 | down |
| *MMD* | 8016832 | NM_012329 // MMD // monocyte to macrophage differentiation-associated // 17q // | NM_012329 | -1,5979 | down |
| *INHBA* | 8139207 | NM_002192 // INHBA // inhibin, beta A // 7p15-p13 // 3624 /// ENST00000242208 // | NM_002192 | -1,59102 | down |
| *DIRC2* | 8082120 | NM_032839 // DIRC2 // disrupted in renal carcinoma 2 // 3q21.1 // 84925 /// ENST | NM_032839 | -1,59091 | down |
| *H2AFB1* | 8171026 | NM_001017990 // H2AFB1 // H2A histone family, member B1 // Xq28 // 474382 /// NM | NM_001017990 | -1,58686 | down |
| *RHOBTB3* | 8106986 | NM_014899 // RHOBTB3 // Rho-related BTB domain containing 3 // 5q15 // 22836 /// | NM_014899 | -1,58068 | down |
| *CCDC113* | 7996198 | NM_014157 // CCDC113 // coiled-coil domain containing 113 // 16q21 // 29070 /// | NM_014157 | -1,57607 | down |
| *TGFBR1* | 8156826 | NM_004612 // TGFBR1 // transforming growth factor, beta receptor 1 // 9q22 // 70 | NM_004612 | -1,57449 | down |
| *KRT81* | 7963353 | NM_002281 // KRT81 // keratin 81 // 12q13 // 3887 /// ENST00000327741 // KRT81 / | NM_002281 | -1,56782 | down |
| *ITPR3* | 8118734 | NM_002224 // ITPR3 // inositol 1,4,5-triphosphate receptor, type 3 // 6p21 // 37 | NM_002224 | -1,565 | down |
| *RAB2A* | 8146564 | NM_002865 // RAB2A // RAB2A, member RAS oncogene family // 8q12.1 // 5862 /// EN | NM_002865 | -1,56498 | down |
| *BEAN1* | 7996281 | NM_001178020 // BEAN1 // brain expressed, associated with NEDD4, 1 // 16q21 // 1 | NM_001178020 | -1,56062 | down |
| *FNTB* | 7975121 | NM_002028 // FNTB // farnesyltransferase, CAAX box, beta // 14q23-q24 // 2342 // | NM_002028 | -1,55939 | down |
| *TMTC3* | 7957478 | NM_181783 // TMTC3 // transmembrane and tetratricopeptide repeat containing 3 // | NM_181783 | -1,5563 | down |
| *LAMB3* | 7924029 | NM_001017402 // LAMB3 // laminin, beta 3 // 1q32 // 3914 /// NM_001127641 // LAM | NM_001017402 | -1,55369 | down |
| *H2AFB1* | 8170994 | NM_001017990 // H2AFB1 // H2A histone family, member B1 // Xq28 // 474382 /// NM | NM_001017990 | -1,55336 | down |
| *H2AFB1* | 8176249 | NM_001017990 // H2AFB1 // H2A histone family, member B1 // Xq28 // 474382 /// NM | NM_001017990 | -1,55336 | down |
| *IL6ST* | 8112139 | NM_002184 // IL6ST // interleukin 6 signal transducer (gp130, oncostatin M recep | NM_002184 | -1,54805 | down |
| *RAB11FIP2* | 7936567 | NM_014904 // RAB11FIP2 // RAB11 family interacting protein 2 (class I) // 10q26. | NM_014904 | -1,5479 | down |
| *RAB12* | 8020029 | NM_001025300 // RAB12 // RAB12, member RAS oncogene family // 18p11.22 // 201475 | NM_001025300 | -1,54771 | down |
| *SIK1* | 8070665 | NM_173354 // SIK1 // salt-inducible kinase 1 // 21q22.3 // 150094 /// ENST000002 | NM_173354 | -1,54668 | down |
| *COPS7A* | 7953395 | NM_001164094 // COPS7A // COP9 constitutive photomorphogenic homolog subunit 7A | NM_001164094 | -1,54661 | down |
| *FAM98A* | 8051413 | NM_015475 // FAM98A // family with sequence similarity 98, member A // 2p22.3 // | NM_015475 | -1,54173 | down |
| *TWSG1* | 8020090 | NM_020648 // TWSG1 // twisted gastrulation homolog 1 (Drosophila) // 18p11.3 // | NM_020648 | -1,53824 | down |
| *SERPINE1* | 8135069 | NM_000602 // SERPINE1 // serpin peptidase inhibitor, clade E (nexin, plasminogen | NM_000602 | -1,53478 | down |
| *ARRDC4* | 7986350 | NM_183376 // ARRDC4 // arrestin domain containing 4 // 15q26.3 // 91947 /// ENST | NM_183376 | -1,53247 | down |
| *STX12* | 7899361 | NM_177424 // STX12 // syntaxin 12 // 1p35.3 // 23673 /// ENST00000373943 // STX1 | NM_177424 | -1,53136 | down |
| *DYNC1LI2* | 8001841 | NM_006141 // DYNC1LI2 // dynein, cytoplasmic 1, light intermediate chain 2 // 16 | NM_006141 | -1,52517 | down |
| *PTX3* | 8083594 | NM_002852 // PTX3 // pentraxin 3, long // 3q25 // 5806 /// ENST00000295927 // PT | NM_002852 | -1,52516 | down |
| *E2F5* | 8147101 | NM_001951 // E2F5 // E2F transcription factor 5, p130-binding // 8q21.2 // 1875 | NM_001951 | -1,52473 | down |
| *DKK1* | 7927631 | NM_012242 // DKK1 // dickkopf homolog 1 (Xenopus laevis) // 10q11.2 // 22943 /// | NM_012242 | -1,52379 | down |
| *CHRDL1* | 8174513 | NM_145234 // CHRDL1 // chordin-like 1 // Xq23 // 91851 /// NM_001143981 // CHRDL | NM_145234 | -1,51848 | down |
| *FAM99A* | 7945660 | NR_026643 // FAM99A // family with sequence similarity 99, member A // 11p15.5 / | NR_026643 | -1,51313 | down |
| *IL32* | 7992828 | NM_001012631 // IL32 // interleukin 32 // 16p13.3 // 9235 /// NM_004221 // IL32 | NM_001012631 | -1,5097 | down |
| *MRFAP1* | 8093936 | NM_033296 // MRFAP1 // Mof4 family associated protein 1 // 4p16.1 // 93621 /// E | NM_033296 | -1,50783 | down |
| *CTTN* | 7942204 | NM_005231 // CTTN // cortactin // 11q13 // 2017 /// NM_138565 // CTTN // cortact | NM_005231 | -1,50779 | down |
| *EREG* | 8095728 | NM_001432 // EREG // epiregulin // 4q13.3 // 2069 /// ENST00000244869 // EREG // | NM_001432 | -1,50629 | down |
| *IGFBP1* | 8132694 | NM_000596 // IGFBP1 // insulin-like growth factor binding protein 1 // 7p13-p12 | NM_000596 | -1,50505 | down |
| *PCGF6* | 8027330 | NM_001011663 // PCGF6 // polycomb group ring finger 6 // 10q24.33 // 84108 /// N | NM_001011663 | -1,50398 | down |
| *NR2F6* | 8035249 | NM_005234 // NR2F6 // nuclear receptor subfamily 2, group F, member 6 // 19p13.1 | NM_005234 | -1,50104 | down |
| *LOC400590* | 8006237 | BC062632 // LOC400590 // hypothetical LOC400590 // 17q11.2 // 400590 /// XR_1094 | BC062632 | 1,50629 | up |
| *MOXD1* | 8129573 | NM_015529 // MOXD1 // monooxygenase, DBH-like 1 // 6q23.2 // 26002 /// ENST00000 | NM_015529 | 1,50958 | up |
| *KIAA1432* | 8154254 | NM_020829 // KIAA1432 // KIAA1432 // 9p24.1 // 57589 /// NM_001135920 // KIAA143 | NM_020829 | 1,51905 | up |
| *DAAM2* | 8119357 | NM_015345 // DAAM2 // dishevelled associated activator of morphogenesis 2 // 6p2 | NM_015345 | 1,51954 | up |
| *TINAG* | 8120315 | NM_014464 // TINAG // tubulointerstitial nephritis antigen // 6p12.1 // 27283 // | NM_014464 | 1,52166 | up |
| *CTDSP2* | 7964579 | NM_005730 // CTDSP2 // CTD (carboxy-terminal domain, RNA polymerase II, polypept | NM_005730 | 1,52333 | up |
| *TMOD1* | 8156706 | NM_003275 // TMOD1 // tropomodulin 1 // 9q22.3 // 7111 /// NM_001166116 // TMOD1 | NM_003275 | 1,52338 | up |
| *PRKD1* | 7978407 | NM_002742 // PRKD1 // protein kinase D1 // 14q11 // 5587 /// ENST00000331968 // | NM_002742 | 1,52505 | up |
| *SHROOM3* | 8095834 | NM_020859 // SHROOM3 // shroom family member 3 // 4q21.1 // 57619 /// ENST000002 | NM_020859 | 1,52549 | up |
| *SP140* | 8048898 | NM_007237 // SP140 // SP140 nuclear body protein // 2q37.1 // 11262 /// NM_00100 | NM_007237 | 1,52552 | up |
| *ESRP1* | 8147351 | NM_017697 // ESRP1 // epithelial splicing regulatory protein 1 // 8q22.1 // 5484 | NM_017697 | 1,52617 | up |
| *SNORD58A* | 8023259 | NR_002571 // SNORD58A // small nucleolar RNA, C/D box 58A // 18q21 // 26791 | NR_002571 | 1,52682 | up |
| *FXYD2* | 7944147 | NM_001680 // FXYD2 // FXYD domain containing ion transport regulator 2 // 11q23 | NM_001680 | 1,52778 | up |
| *MATN2* | 8147516 | NM_002380 // MATN2 // matrilin 2 // 8q22 // 4147 /// NM_030583 // MATN2 // matri | NM_002380 | 1,53026 | up |
| *BTN3A1* | 8117458 | NM_001145009 // BTN3A1 // butyrophilin, subfamily 3, member A1 // 6p22.1 // 1111 | NM_001145009 | 1,53043 | up |
| *SORBS1* | 7935188 | NM_001034954 // SORBS1 // sorbin and SH3 domain containing 1 // 10q23.33 // 1058 | NM_001034954 | 1,53267 | up |
| *KRT80* | 7963333 | NM_182507 // KRT80 // keratin 80 // 12q13.13 // 144501 /// NM_001081492 // KRT80 | NM_182507 | 1,534 | up |
| *WDR91* | 8143070 | NM_014149 // WDR91 // WD repeat domain 91 // 7q33 // 29062 /// ENST00000354475 / | NM_014149 | 1,53751 | up |
| *SIRPD* | 8064464 | NM_178460 // SIRPD // signal-regulatory protein delta // 20p13 // 128646 /// ENS | NM_178460 | 1,53778 | up |
| *SERTAD4* | 7909503 | NM_019605 // SERTAD4 // SERTA domain containing 4 // 1q32.1-q41 // 56256 /// ENS | NM_019605 | 1,53877 | up |
| *NFKBIZ* | 8081386 | NM_031419 // NFKBIZ // nuclear factor of kappa light polypeptide gene enhancer i | NM_031419 | 1,53886 | up |
| *FCGBP* | 8036787 | NM_003890 // FCGBP // Fc fragment of IgG binding protein // 19q13.1 // 8857 /// | NM_003890 | 1,5434 | up |
| *IL23A* | 7956251 | NM_016584 // IL23A // interleukin 23, alpha subunit p19 // 12q13.3 // 51561 /// | NM_016584 | 1,54902 | up |
| *IL7* | 8151447 | NM_000880 // IL7 // interleukin 7 // 8q12-q13 // 3574 /// ENST00000263851 // IL7 | NM_000880 | 1,54992 | up |
| *FAM126B* | 8058182 | NM_173822 // FAM126B // family with sequence similarity 126, member B // 2q33.1 | NM_173822 | 1,55361 | up |
| *STYK1* | 7961215 | NM_018423 // STYK1 // serine/threonine/tyrosine kinase 1 // 12p13.2 // 55359 /// | NM_018423 | 1,55644 | up |
| *APOL3* | 8075695 | NR_027833 // APOL3 // apolipoprotein L, 3 // 22q13.1 // 80833 /// NR_027834 // A | NR_027833 | 1,55678 | up |
| *SLC48A1* | 7955055 | NM_017842 // SLC48A1 // solute carrier family 48 (heme transporter), member 1 // | NM_017842 | 1,56242 | up |
| *ANKRD36BP1* | 7922121 | NR_026844 // ANKRD36BP1 // ankyrin repeat domain 36B pseudogene 1 // 1q24.2 // 8 | NR_026844 | 1,56662 | up |
| *KCNN3* | 7920552 | NM_002249 // KCNN3 // potassium intermediate/small conductance calcium-activated | NM_002249 | 1,56685 | up |
| *SEC24C* | 7928369 | NM_004922 // SEC24C // SEC24 family, member C (S. cerevisiae) // 10q22.2 // 9632 | NM_004922 | 1,5669 | up |
| *FAM111A* | 7940153 | NM_022074 // FAM111A // family with sequence similarity 111, member A // 11q12.1 | NM_022074 | 1,56861 | up |
| *CDRT1* | 8012951 | NM_006382 // CDRT1 // CMT1A duplicated region transcript 1 // 17p12 // 374286 // | NM_006382 | 1,57108 | up |
| *PCDH18* | 8102792 | NM_019035 // PCDH18 // protocadherin 18 // 4q31 // 54510 /// ENST00000344876 // | NM_019035 | 1,57237 | up |
| *FAM83B* | 8120335 | NM_001010872 // FAM83B // family with sequence similarity 83, member B // 6p12.1 | NM_001010872 | 1,5735 | up |
| *SECTM1* | 8019486 | NM_003004 // SECTM1 // secreted and transmembrane 1 // 17q25 // 6398 /// ENST000 | NM_003004 | 1,57589 | up |
| *APOBEC3F* | 8073081 | NM_145298 // APOBEC3F // apolipoprotein B mRNA editing enzyme, catalytic polypep | NM_145298 | 1,57847 | up |
| *TNFSF18* | 7922337 | NM_005092 // TNFSF18 // tumor necrosis factor (ligand) superfamily, member 18 // | NM_005092 | 1,58058 | up |
| *GIMAP2* | 8137250 | NM_015660 // GIMAP2 // GTPase, IMAP family member 2 // 7q36.1 // 26157 /// ENST0 | NM_015660 | 1,5823 | up |
| *ABCC2* | 7929779 | NM_000392 // ABCC2 // ATP-binding cassette, sub-family C (CFTR/MRP), member 2 // | NM_000392 | 1,58496 | up |
| *IL1R2* | 8043981 | NM_004633 // IL1R2 // interleukin 1 receptor, type II // 2q12 // 7850 /// NM_173 | NM_004633 | 1,58652 | up |
| *GBP3* | 7917503 | NM_018284 // GBP3 // guanylate binding protein 3 // 1p22.2 // 2635 /// ENST00000 | NM_018284 | 1,58717 | up |
| *RARRES3* | 7940775 | NM_004585 // RARRES3 // retinoic acid receptor responder (tazarotene induced) 3 | NM_004585 | 1,58932 | up |
| *NID1* | 7925320 | NM_002508 // NID1 // nidogen 1 // 1q43 // 4811 /// ENST00000264187 // NID1 // ni | NM_002508 | 1,59796 | up |
| *GJB7* | 8128007 | NM_198568 // GJB7 // gap junction protein, beta 7, 25kDa // 6q15 // 375519 /// E | NM_198568 | 1,59908 | up |
| *SNX29* | 7993281 | NM_001080530 // SNX29 // sorting nexin 29 // 16p13.13-p13.12 // 92017 /// ENST00 | NM_001080530 | 1,60109 | up |
| *SLC6A6* | 8078014 | NM_003043 // SLC6A6 // solute carrier family 6 (neurotransmitter transporter, ta | NM_003043 | 1,60147 | up |
| *PRSS2* | 8136807 | NM_002770 // PRSS2 // protease, serine, 2 (trypsin 2) // 7q34 // 5645 /// ENST00 | NM_002770 | 1,60642 | up |
| *APOL2* | 8075720 | NM_030882 // APOL2 // apolipoprotein L, 2 // 22q12 // 23780 /// NM_145637 // APO | NM_030882 | 1,61285 | up |
| *SIRPG* | 8064485 | NM_018556 // SIRPG // signal-regulatory protein gamma // 20p13 // 55423 /// NM_0 | NM_018556 | 1,61595 | up |
| *RAB19* | 8136580 | NM_001008749 // RAB19 // RAB19, member RAS oncogene family // 7q34 // 401409 /// | NM_001008749 | 1,61638 | up |
| *STS* | 8165866 | NM_000351 // STS // steroid sulfatase (microsomal), isozyme S // Xp22.32 // 412 | NM_000351 | 1,61674 | up |
| *IL1B* | 8054722 | NM_000576 // IL1B // interleukin 1, beta // 2q14 // 3553 /// ENST00000263341 // | NM_000576 | 1,61789 | up |
| *GBP4* | 7917561 | NM_052941 // GBP4 // guanylate binding protein 4 // 1p22.2 // 115361 /// ENST000 | NM_052941 | 1,62094 | up |
| *PPP1R3C* | 7934997 | NM_005398 // PPP1R3C // protein phosphatase 1, regulatory (inhibitor) subunit 3C | NM_005398 | 1,62099 | up |
| *C14orf105* | 7979378 | NM_018168 // C14orf105 // chromosome 14 open reading frame 105 // 14q22.3 // 551 | NM_018168 | 1,62319 | up |
| *SP110* | 8059650 | NM_080424 // SP110 // SP110 nuclear body protein // 2q37.1 // 3431 /// NM_004509 | NM_080424 | 1,62478 | up |
| *OAS2* | 7958913 | NM_002535 // OAS2 // 2'-5'-oligoadenylate synthetase 2, 69/71kDa // 12q24.2 // 4 | NM_002535 | 1,62766 | up |
| *VCAN* | 8106743 | NM_004385 // VCAN // versican // 5q14.3 // 1462 /// NM_001164097 // VCAN // vers | NM_004385 | 1,6361 | up |
| *BTN3A3* | 8117476 | NM_006994 // BTN3A3 // butyrophilin, subfamily 3, member A3 // 6p21.3 // 10384 / | NM_006994 | 1,63637 | up |
| *TCN2* | 8072360 | NM_000355 // TCN2 // transcobalamin II // 22q12.2 // 6948 /// NM_001184726 // TC | NM_000355 | 1,64576 | up |
| *AKAP5* | 7975066 | NM_004857 // AKAP5 // A kinase (PRKA) anchor protein 5 // 14q21-q24 // 9495 /// | NM_004857 | 1,65053 | up |
| *CCNE2* | 8151871 | NM_057749 // CCNE2 // cyclin E2 // 8q22.1 // 9134 /// ENST00000308108 // CCNE2 / | NM_057749 | 1,65691 | up |
| *ZNF528* | 8030931 | NM_032423 // ZNF528 // zinc finger protein 528 // 19q13 // 84436 /// ENST0000036 | NM_032423 | 1,66485 | up |
| *C2orf15* | 8043835 | BC021264 // C2orf15 // chromosome 2 open reading frame 15 // 2q11.2 // 150590 // | BC021264 | 1,66699 | up |
| *STX3* | 7940191 | NM_004177 // STX3 // syntaxin 3 // 11q12.1 // 6809 /// NM_001178040 // STX3 // s | NM_004177 | 1,6696 | up |
| *SAA2* | 7946983 | NM_030754 // SAA2 // serum amyloid A2 // 11p15.1-p14 // 6289 /// BC020795 // SAA | NM_030754 | 1,67093 | up |
| *AGR2* | 8138381 | NM_006408 // AGR2 // anterior gradient homolog 2 (Xenopus laevis) // 7p21.3 // 1 | NM_006408 | 1,67344 | up |
| *IL1R1* | 8043995 | NM_000877 // IL1R1 // interleukin 1 receptor, type I // 2q12 // 3554 /// ENST000 | NM_000877 | 1,68069 | up |
| *FAS* | 7929032 | NM_000043 // FAS // Fas (TNF receptor superfamily, member 6) // 10q24.1 // 355 / | NM_000043 | 1,68122 | up |
| *PDZK1IP1* | 7915910 | NM_005764 // PDZK1IP1 // PDZK1 interacting protein 1 // 1p33 // 10158 /// ENST00 | NM_005764 | 1,69433 | up |
| *RRAGC* | 7915160 | NM_022157 // RRAGC // Ras-related GTP binding C // 1p34 // 64121 /// ENST0000037 | NM_022157 | 1,7052 | up |
| *DNAH14* | 7910047 | NM_001373 // DNAH14 // dynein, axonemal, heavy chain 14 // 1q42.12 // 127602 /// | NM_001373 | 1,7103 | up |
| *ANXA4* | 8042468 | NM_001153 // ANXA4 // annexin A4 // 2p13 // 307 /// ENST00000394295 // ANXA4 // | NM_001153 | 1,71089 | up |
| *TRANK1* | 8086125 | NM_014831 // TRANK1 // tetratricopeptide repeat and ankyrin repeat containing 1 | NM_014831 | 1,71931 | up |
| *LIPC* | 7983928 | NM_000236 // LIPC // lipase, hepatic // 15q21-q23 // 3990 /// ENST00000299022 // | NM_000236 | 1,72544 | up |
| *HIPK3* | 7939197 | NM_005734 // HIPK3 // homeodomain interacting protein kinase 3 // 11p13 // 10114 | NM_005734 | 1,73566 | up |
| *IFI44* | 7902553 | NM_006417 // IFI44 // interferon-induced protein 44 // 1p31.1 // 10561 /// ENST0 | NM_006417 | 1,73934 | up |
| *TLR3* | 8098611 | NM_003265 // TLR3 // toll-like receptor 3 // 4q35 // 7098 /// ENST00000296795 // | NM_003265 | 1,74161 | up |
| *IFIT3* | 7929052 | NM_001031683 // IFIT3 // interferon-induced protein with tetratricopeptide repea | NM_001031683 | 1,74378 | up |
| *SFT2D2* | 7907135 | NM_199344 // SFT2D2 // SFT2 domain containing 2 // 1q24.2 // 375035 /// ENST0000 | NM_199344 | 1,7479 | up |
| *PIK3R3* | 7915787 | NM_003629 // PIK3R3 // phosphoinositide-3-kinase, regulatory subunit 3 (gamma) / | NM_003629 | 1,75558 | up |
| *TCF19* | 8118086 | NM_007109 // TCF19 // transcription factor 19 // 6p21.3 // 6941 /// NM_001077511 | NM_007109 | 1,7578 | up |
| *TCF19* | 8177947 | NM_007109 // TCF19 // transcription factor 19 // 6p21.3 // 6941 /// NM_001077511 | NM_007109 | 1,7578 | up |
| *STON2* | 7980537 | NM_033104 // STON2 // stonin 2 // 14q31.1 // 85439 /// ENST00000267540 // STON2 | NM_033104 | 1,764 | up |
| *ID2* | 8040103 | NM_002166 // ID2 // inhibitor of DNA binding 2, dominant negative helix-loop-hel | NM_002166 | 1,76707 | up |
| *GPR116* | 8126798 | NM_015234 // GPR116 // G protein-coupled receptor 116 // 6p12.3 // 221395 /// NM | NM_015234 | 1,77121 | up |
| *RAB37* | 8009666 | NM_175738 // RAB37 // RAB37, member RAS oncogene family // 17q25.1 // 326624 /// | NM_175738 | 1,77275 | up |
| *TCF19* | 8179228 | NM_007109 // TCF19 // transcription factor 19 // 6p21.3 // 6941 /// NM_001077511 | NM_007109 | 1,77289 | up |
| *GPR81* | 7967325 | NM_032554 // GPR81 // G protein-coupled receptor 81 // 12q24.31 // 27198 /// ENS | NM_032554 | 1,78372 | up |
| *LOC613266* | 8065084 | AK125594 // LOC613266 // hypothetical LOC613266 // 20p12.1 // 613266 | AK125594 | 1,79636 | up |
| *LCP1* | 7971461 | NM_002298 // LCP1 // lymphocyte cytosolic protein 1 (L-plastin) // 13q14.3 // 39 | NM_002298 | 1,80347 | up |
| *OASL* | 7967117 | NM_003733 // OASL // 2'-5'-oligoadenylate synthetase-like // 12q24.2 // 8638 /// | NM_003733 | 1,80811 | up |
| *IFIT1* | 7929065 | NM_001548 // IFIT1 // interferon-induced protein with tetratricopeptide repeats | NM_001548 | 1,82061 | up |
| *OAS1* | 7958884 | NM_016816 // OAS1 // 2',5'-oligoadenylate synthetase 1, 40/46kDa // 12q24.1 // 4 | NM_016816 | 1,8414 | up |
| *ATP6V0D2* | 8147145 | NM_152565 // ATP6V0D2 // ATPase, H+ transporting, lysosomal 38kDa, V0 subunit d2 | NM_152565 | 1,85428 | up |
| *KITLG* | 7965322 | NM_000899 // KITLG // KIT ligand // 12q22 // 4254 /// NM_003994 // KITLG // KIT | NM_000899 | 1,85914 | up |
| *RNF152* | 8023598 | NM_173557 // RNF152 // ring finger protein 152 // 18q21.33 // 220441 /// ENST000 | NM_173557 | 1,86422 | up |
| *TSPAN8* | 7964927 | NM_004616 // TSPAN8 // tetraspanin 8 // 12q14.1-q21.1 // 7103 /// ENST0000024782 | NM_004616 | 1,86749 | up |
| *SAMD9* | 8140967 | NM_017654 // SAMD9 // sterile alpha motif domain containing 9 // 7q21.2 // 54809 | NM_017654 | 1,87125 | up |
| *RSAD2* | 8040080 | NM_080657 // RSAD2 // radical S-adenosyl methionine domain containing 2 // 2p25. | NM_080657 | 1,878 | up |
| *SAMD9L* | 8140971 | NM_152703 // SAMD9L // sterile alpha motif domain containing 9-like // 7q21.2 // | NM_152703 | 1,89104 | up |
| *ID3* | 7913655 | NM_002167 // ID3 // inhibitor of DNA binding 3, dominant negative helix-loop-hel | NM_002167 | 1,90123 | up |
| *SCNN1A* | 7960529 | NM_001038 // SCNN1A // sodium channel, nonvoltage-gated 1 alpha // 12p13 // 6337 | NM_001038 | 1,90858 | up |
| *APOBEC3G* | 8073088 | NM_021822 // APOBEC3G // apolipoprotein B mRNA editing enzyme, catalytic polypep | NM_021822 | 1,90884 | up |
| *TSPAN1* | 7901175 | NM_005727 // TSPAN1 // tetraspanin 1 // 1p34.1 // 10103 /// ENST00000372003 // T | NM_005727 | 1,91032 | up |
| *IFIT2* | 7929047 | NM_001547 // IFIT2 // interferon-induced protein with tetratricopeptide repeats | NM_001547 | 1,91049 | up |
| *CD96* | 8081564 | NM_198196 // CD96 // CD96 molecule // 3q13.13-q13.2 // 10225 /// NM_005816 // CD | NM_198196 | 1,9284 | up |
| *UCA1* | 8026490 | NR_015379 // UCA1 // urothelial cancer associated 1 (non-protein coding) // 19p1 | NR_015379 | 1,94931 | up |
| *B3GALT5* | 8068633 | NM_033171 // B3GALT5 // UDP-Gal:betaGlcNAc beta 1,3-galactosyltransferase, polyp | NM_033171 | 1,95858 | up |
| *CEACAM1* | 8037205 | NM_001712 // CEACAM1 // carcinoembryonic antigen-related cell adhesion molecule | NM_001712 | 1,99498 | up |
| *ADAMTS9* | 8088560 | NM_182920 // ADAMTS9 // ADAM metallopeptidase with thrombospondin type 1 motif, | NM_182920 | 2,00628 | up |
| *GPR110* | 8126820 | NM_153840 // GPR110 // G protein-coupled receptor 110 // 6p12.3 // 266977 /// NM | NM_153840 | 2,02455 | up |
| *BCMO1* | 7997401 | NM_017429 // BCMO1 // beta-carotene 15,15'-monooxygenase 1 // 16q23.2 // 53630 / | NM_017429 | 2,03408 | up |
| *FXYD2* | 7951966 | NM_001127489 // FXYD2 // FXYD domain containing ion transport regulator 2 // 11q | NM_001127489 | 2,04434 | up |
| *KCNJ16* | 8009493 | NM_170742 // KCNJ16 // potassium inwardly-rectifying channel, subfamily J, membe | NM_170742 | 2,04693 | up |
| *PTGS1* | 8157650 | NM_000962 // PTGS1 // prostaglandin-endoperoxide synthase 1 (prostaglandin G/H s | NM_000962 | 2,04999 | up |
| *LOC388022* | 7977270 | AK131040 // LOC388022 // hypothetical LOC388022 // 14q32.33 // 388022 | AK131040 | 2,12178 | up |
| *NPNT* | 8096704 | NM_001184690 // NPNT // nephronectin // 4q24 // 255743 /// NM_001033047 // NPNT | NM_001184690 | 2,1625 | up |
| *ABCA12* | 8058708 | NM_173076 // ABCA12 // ATP-binding cassette, sub-family A (ABC1), member 12 // 2 | NM_173076 | 2,22558 | up |
| *STC1* | 8149825 | NM_003155 // STC1 // stanniocalcin 1 // 8p21-p11.2 // 6781 /// ENST00000290271 / | NM_003155 | 2,27174 | up |
| *MPZL2* | 7952046 | NM_144765 // MPZL2 // myelin protein zero-like 2 // 11q24 // 10205 /// NM_005797 | NM_144765 | 2,27354 | up |
| *ASB2* | 7981020 | NM_016150 // ASB2 // ankyrin repeat and SOCS box-containing 2 // 14q31-q32 // 51 | NM_016150 | 2,2865 | up |
| *SERPINB2* | 8021635 | NM_001143818 // SERPINB2 // serpin peptidase inhibitor, clade B (ovalbumin), mem | NM_001143818 | 2,29768 | up |
| *GAL3ST1* | 8075401 | NM_004861 // GAL3ST1 // galactose-3-O-sulfotransferase 1 // 22q12.2 // 9514 /// | NM_004861 | 2,31624 | up |
| *ALPK2* | 8023528 | NM_052947 // ALPK2 // alpha-kinase 2 // 18q21.31 // 115701 /// ENST00000361673 / | NM_052947 | 2,39344 | up |
| *CD177* | 8029280 | NM_020406 // CD177 // CD177 molecule // 19q13.2 // 57126 /// ENST00000457794 // | NM_020406 | 2,45951 | up |
| *SIRPB1* | 8064471 | NM_006065 // SIRPB1 // signal-regulatory protein beta 1 // 20p13 // 10326 /// NM | NM_006065 | 2,57732 | up |
| *SGK2* | 8062728 | NM_016276 // SGK2 // serum/glucocorticoid regulated kinase 2 // 20q13.2 // 10110 | NM_016276 | 2,71153 | up |
| *PDIA6* | 8040249 | NM_005742 // PDIA6 // protein disulfide isomerase family A, member 6 // 2p25.1 / | NM_005742 | 2,84388 | up |
| *CD177* | 8037298 | NM_020406 // CD177 // CD177 molecule // 19q13.2 // 57126 /// ENST00000457794 // | NM_020406 | 3,06146 | up |
| *SERPINA6* | 7981059 | NM_001756 // SERPINA6 // serpin peptidase inhibitor, clade A (alpha-1 antiprotei | NM_001756 | 3,28921 | up |
